# Differential Network Analysis Reveals Evolutionary Complexity in Secondary Metabolism of *Rauvolfia serpentina* over *Catharanthus roseus*

**DOI:** 10.3389/fpls.2016.01229

**Published:** 2016-08-18

**Authors:** Shivalika Pathania, Ganesh Bagler, Paramvir S. Ahuja

**Affiliations:** ^1^Biotechnology Division, CSIR-Institute of Himalayan Bioresource Technology, Council of Scientific and Industrial ResearchPalampur, India; ^2^Center for Computational Biology, Indraprastha Institute of Information Technology Delhi (IIIT-Delhi)New Delhi, India; ^3^Centre for Biologically Inspired System Science, Indian Institute of Technology JodhpurJodhpur, India; ^4^Dhirubhai Ambani Institute of Information and Communication TechnologyGandhinagar, India; ^5^Indian Institute of Science Education and Research (IISER) MohaliMohali, India

**Keywords:** comparative network analysis, plant systems biology, secondary metabolism and enzymes, *Rauvolfia serpentina*, *Catharanthus roseus*

## Abstract

Comparative co-expression analysis of multiple species using high-throughput data is an integrative approach to determine the uniformity as well as diversification in biological processes. *Rauvolfia serpentina* and *Catharanthus roseus*, both members of Apocyanacae family, are reported to have remedial properties against multiple diseases. Despite of sharing upstream of terpenoid indole alkaloid pathway, there is significant diversity in tissue-specific synthesis and accumulation of specialized metabolites in these plants. This led us to implement comparative co-expression network analysis to investigate the modules and genes responsible for differential tissue-specific expression as well as species-specific synthesis of metabolites. Toward these goals differential network analysis was implemented to identify candidate genes responsible for diversification of metabolites profile. Three genes were identified with significant difference in connectivity leading to differential regulatory behavior between these plants. These genes may be responsible for diversification of secondary metabolism, and thereby for species-specific metabolite synthesis. The network robustness of *R. serpentina*, determined based on topological properties, was also complemented by comparison of gene-metabolite networks of both plants, and may have evolved to have complex metabolic mechanisms as compared to *C. roseus* under the influence of various stimuli. This study reveals evolution of complexity in secondary metabolism of *R. serpentina*, and key genes that contribute toward diversification of specific metabolites.

## Introduction

Comparative analysis is implemented for comparing two or more organisms to identify their similarities as well as mechanisms for diversification toward various key biological processes such as reproduction, defense response, metabolic pathways, photosynthesis, and many more. It involves comparison of either different species of same family or different taxonomic organisms of same species (Wei et al., 2002). It works on the assumption that biologically relevant processes remain conserved across organisms as compared to irrelevant associations which wither away over evolutionary time scale (Hansen et al., 2014). Comparative genomics (Wei et al., 2002), comparative analysis of protein domain organization (Ye and Godzik, 2004), phylogenetic comparative methods (Lavin et al., 2008), and comparative co-expression analysis (Alter et al., 2003; Hansen et al., 2014) are a few methods to compare different aspects of multiple organisms. Comparative genomics has been reported to be one of the most successful methods used for characterization of gene functions, biomarkers, and to investigate evolutionary history in humans (Moreno et al., 2008) as well as plants (Moreno et al., 2008; Michael and Jackson, 2013). Currently, genomes of only 55 plants have been annotated which mainly include model plants and crop plants (Michael and Jackson, 2013). There is still a substantial lacuna of genomes of non-model plants making comparative genomics unsuitable to reveal functionality of genes involved in specific biological processes and their inter-relationships. However, comparative co-expression analysis overcomes this drawback, and has been successfully implemented in identification of cis-regulatory motifs, functions of unknown genes, and diversity in various biological pathways across different organisms (Emmert-Streib, 2007; Hansen et al., 2014).

Co-expression represents a coordinated behavior among genes across a variety of experimental conditions, and therefore often indicates functional associations among them (Usadel et al., 2009). Based on this understanding, various studies have implemented co-expression network analysis approach to perform functional characterization of genes (Ma and Peng, 2006; Aoki et al., 2007; Romero-Campero et al., 2013; Liang et al., 2014; Li et al., 2015; Ransbotyn et al., 2015; Wilson et al., 2015). Comparative co-expression analysis has also been known to reduce the rate of false positives since non-relevant patterns are less likely to be reproduced multiple times in co-expression network of different organisms (Hansen et al., 2014). This analysis also presents one of the highly robust methods to perform inter-species or multi-species comparison, by assigning functional information from model organism, to determine genes involved in core as well as diversified pathways (Oldham et al., 2006; Emmert-Streib, 2007; Miller et al., 2010; Hansen et al., 2014).

*Rauvolfia serpentina* is an important medicinal plant of Apocynaceae family that is endemic to Indian subcontinent and South-East Asian countries, and is reported to be present in the Himalayan region distributed over the foothills up to elevations of 1300–1400 m (Dey and De, 2011). This plant produces natural molecules of remedial properties which are used in the treatment of various diseases such as hypertension, diabetes, and ventricular arrhythmias (Lelek and Furedi Szabo, 1961; Nammi et al., 2005; Jerie, 2007; Dey and De, 2011). Reserpine is the principle component of *R. serpentina* which is used to treat hypertension (Nammi et al., 2005), tachycardia (Jerie, 2007), and allergy (Lelek and Furedi Szabo, 1961). Other compounds such as ajmaline (Köppel et al., 1989), serpentine (Beljanski and Beljanski, 1982), rescinnamine (Nammi et al., 2005), and yohimbine (Singh et al., 2004) are also used as therapeutics in the treatment of different diseases. *Catharanthus roseus*, a closely related medicinal plant to *R. serpentina* of same family, is also known for its anti-cancerous properties, where vinblastine and vincristine are the most important molecules that are effectively used in treatment of several cancers (van Der Heijden et al., 2004).

*C. roseus*, a model plant of Apocynaceae family, has different spectrum of important terpenoid indole alkaloids (TIAs) in aerial (Zhong et al., 2010) as compared to underground tissues in *R. serpentina* (Lelek and Furedi Szabo, 1961; Nammi et al., 2005; Jerie, 2007; Dey and De, 2011). Major phytochemical constituents of *R. serpentina* are root indole alkaloids (Pathania et al., 2013), whereas in *C. roseus*, antineoplastic bisindole alkaloids are principal compounds that are restricted mainly to aerial parts (Zhong et al., 2010; Zhu et al., 2015). Moreover, synthesis of such secondary metabolites is compartmentalized into specific tissues (Shukla et al., 2006). Although, these closely related medicinal plants are reported to share upstream of TIA pathway (O'Connor and Maresh, 2006), there is significant diversity in important secondary metabolites at downstream of this pathway (van Der Heijden et al., 2004). Genes responsible for this differential tissue-specific as well as species-specific synthesis of metabolites can be determined using comparative co-expression analysis approach. Differential analysis provides a list of genes that are not only differentially expressed but are highly connected as well. This difference in connectivity also coincides with rewiring of interactions in biological pathways that leads to speciation (Oldham et al., 2006). Knowing that majority of pathways and regulatory mechanisms are similar between these plants, characterization of candidate genes with significant differences in their expression is crucial to determine pathways responsible for tissue-specific synthesis and accumulation of major secondary metabolites.

Advances in release of genome-wide gene expression data has allowed researchers to investigate various biological processes, their regulatory mechanisms, and inter-species comparisons using network biology approach. High-throughput next generation sequencing data of various model plants (Michael and Jackson, 2013) has been utilized to obtain network models using different classical approaches (Emmert-Streib et al., 2012). Fortunately, large-scale transcriptomics data for various medicinal plants, including *R. serpentina* and *C. roseus*, is also available at Medicinal Plant Genomics resource (MPGR, http://medicinalplantgenomics.msu.edu/) (Góngora-Castillo et al., 2012). Therefore, availability of such high-throughput expression data and provision for their integration with computational bioinformatics analysis prompted us to identify candidate genes/biomarkers responsible for the difference in spectrum of indole alkaloids between *R. serpentina* and *C. roseus*. Various traditional methods have been used to compare these closely related plants that work on small-scale (Gerasimenko et al., 2002). In our earlier study, a graph theoretical approach has been implemented to identify transcription factors (TFs) involved in regulation of secondary metabolism of *R. serpentina* (Pathania and Acharya, 2016), but to the best of our knowledge, comparative investigation of these plants using network-based approach is hitherto not undertaken. Integration of graph theory based approach and “-*omics*” data have facilitated systems-level comparison of underlying molecular pathways to unravel their complexity as well as mechanisms of differential regulation (Emmert-Streib, 2007; Emmert-Streib et al., 2012; Shaik and Ramakrishna, 2013). Comparative co-expression analysis has also been utilized to compare plant systems on the basis of microarray data (Movahedi et al., 2012). Differentially expressed genes (DEGs) have been reported to be involved in secondary metabolite synthesis (Tao et al., 2013; Zhou et al., 2015) that may be responsible for the diverse metabolite synthesis in *R. serpentina* and *C. roseus*.

**Figure 1:**
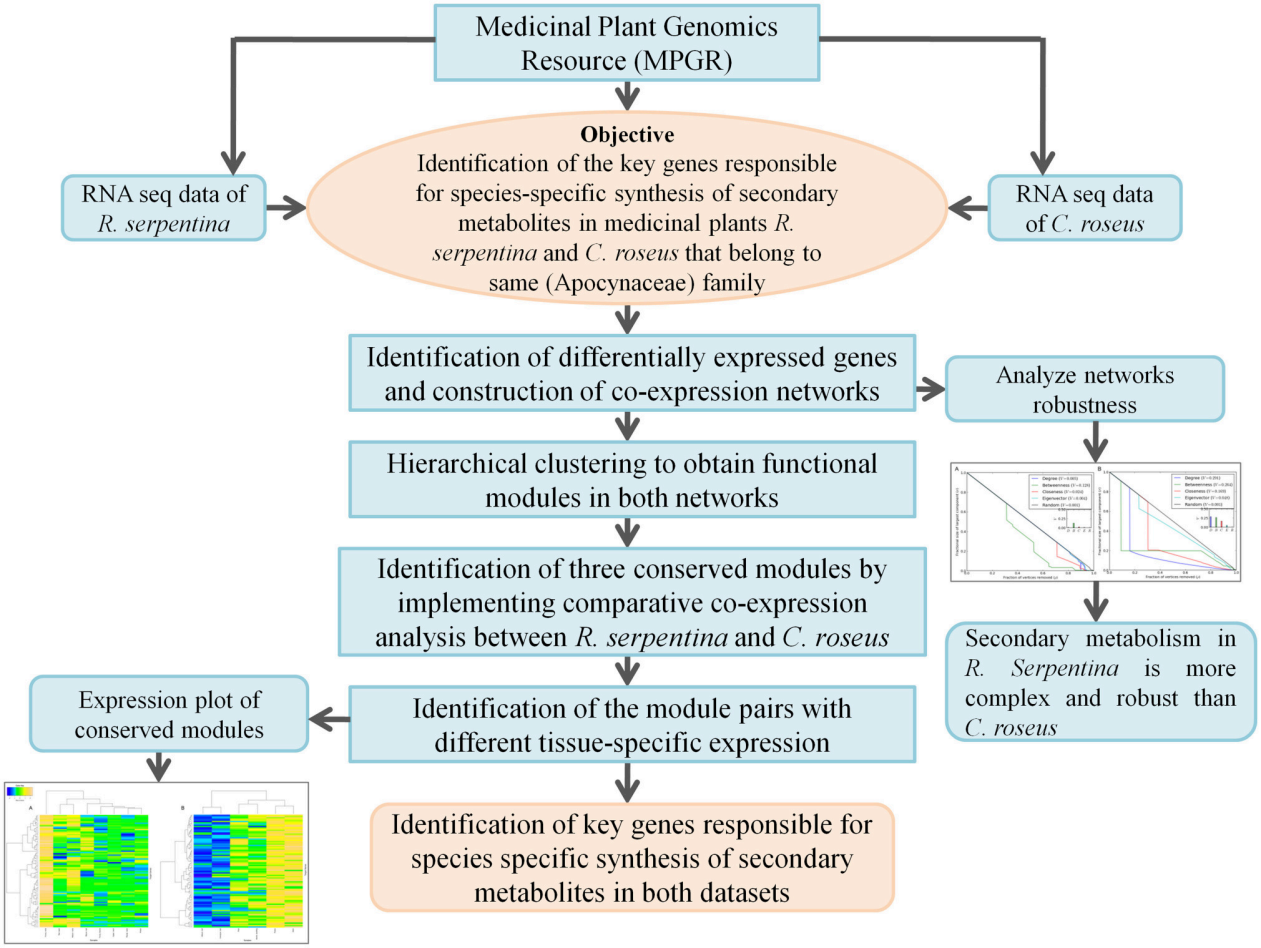
**Strategy implemented to identify genes responsible for species-specific synthesis of metabolites in *R. serpentina* and *C. roses* through comparative co-expression analysis**.

In this study, we implemented a meta-analysis of RNA sequencing data through comparative co-expression analysis between *R. serpentina* and *C. roseus* (Figure 1). In order to determine candidate genes responsible for species-specific synthesis of metabolites in both medicinal plants, differential expression analysis was carried out using network-based approach. Weighted co-expression networks for both datasets were generated from the DEGs obtained. Analysis of topological properties of networks suggested that *R. serpentina* network is more robust and may have evolved to acquire complexity in secondary metabolism to synthesize specific metabolites over *C. roseus* under the influence of external stimuli. A few of the candidate genes obtained were found to be shared between both datasets with significant difference in their intramodular connectivity. This difference in connectivity is responsible for rewiring of interactions, and thereby differential regulatory behavior of both datasets that may led to species-specific synthesis secondary metabolites. The observed robustness of *R. serpentina* network as compared to that of *C. roseus* was also complemented by complexity of its gene-metabolite network which may have evolved due to its complex metabolic mechanisms under the influence of various stimuli. This approach allowed us to determine conserved and diversified pathways as well as candidate genes responsible for species-specific metabolite synthesis.

## Materials and methods

In order to compare *R. serpentina* and *C. roseus*, following protocol was implemented: (1) retrieval and pre-processing of datasets, (2) identification of differentially expressed genes, (3) construction and validation of gene co-expression networks, (4) evaluation of network robustness, (5) functional annotation and enrichment analysis of modules, and (6) comparison of gene co-expression networks.

### Retrieval and pre-processing of datasets

Transcriptomic sequences and expression profile data for different tissue samples of *R. serpentina* (8) and *C. roseus* (6) were retrieved from the Medicinal Plant Genomics Resource database (MPGR, http://medicinalplantgenomics.msu.edu/) (Góngora-Castillo et al., 2012). Transcripts from both datasets were annotated by performing BLASTX (Altschul et al., 1997) search against the reference *Arabidopsis* proteome (TAIR10, http://Arabidopsis.org). An e-value cutoff of 1e-05 was used to identity orthologous genes, and top hit annotations were preserved for further analyses. Expression data (log transformed FPKM values) of different tissues were obtained for *R. serpentina* (mature leaf, young leaves, upper stem, young roots, mature roots, red stem, flower, and woody stem) and *C. roseus* (stem, mature leaf, immature leaf, root, flower, and sterile seedling). For comparative analysis expression data of only common transcripts was considered to construct gene co-expression networks that were further probed to elucidate species-specific regulation of secondary metabolism. As an initial refinement step, genes with excessive missing values and sample outliers were excluded to reduce the noise, leaving behind most informative genes (Miller et al., 2010; Langfelder and Horvath, 2012). Orthologs of *A. thaliana*, expressed in both datasets, were also pre-processed to assess their comparability by computing correlation between their average gene expressions. Transcripts with identical *Arabidopsis* ortholog, in each dataset, were filtered on the basis of standard deviation among samples.

### Identification of differentially expressed genes

Two sample *t*-test is used as a standard measure to compare multiple samples on the basis of difference in variances between datasets. Variance was computed through “genefilter” library of Bioconductor *v* 3.1 package (http://www.bioconductor.org/), and genes with low variance (≤ 30%) were excluded. Variance among samples was computed as follows:

$$varinace=\frac{SD_{1}^{2}\left(n_{1}-1\right)+\;SD_{2}^{2}\;(n_{2}-1)}{n_{1}\;+\;n_{2}\;-2}$$

where *n*_1_ and *n*_2_ are number of observations, and *SD*_1_ and *SD*_2_ are standard deviation of *R. serpentina*, and *C. roseus* datasets, respectively.

The *t*-test was implemented again to select significant genes (McCluskey and Lalkhen, 2007; Zhang et al., 2012). The *p*-values were computed, and further corrected for multiple testing problems using Benjamini and Hochberg method to calculate the false discovery rate (FDR) adjusted *p*-value (*q*-value) (Benjamini and Hochberg, 1995). The genes were considered statistically significant if their *q*-values were ≤0.05. Principal Component Analysis (PCA) was performed to determine overall expression patterns between both datasets.

### Construction and validation of gene co-expression networks

Comparative co-expression network analysis was performed using “Weighted Gene Co-Expression Network Analysis” (WGCNA) library (Langfelder and Horvath, 2008) of R statistical package *v* 3.0.1. After initial data pre-processing, expression values of significant DEGs were used to construct two independent signed networks (networks that preserve the sign of correlations among expression profiles) for both datasets. For each weighted network, Pearson correlation matrices (corresponding to gene expression dataset) were computed, which were further transformed into matrices of connection strengths using a power function (β) that fits best to its scale-free behavior (Zhang and Horvath, 2005). These connection strengths were again transformed into a topological overlap similarity (TOM) measure which was used to compute dissimilarity TOM (DistTOM), a robust measure of pairwise interconnectedness (Yip and Horvath, 2007).

In order to reduce the complexity of whole networks comparison, clustering was performed independently that led to generation of distinct set of grouped genes with similar functional characteristics. Dissimilarity matrices were generated from corresponding TOMs to identify modules through average linkage hierarchical clustering by applying dynamic tree cut algorithm (Langfelder and Horvath, 2008). For this analysis, appropriate deep split was set to obtain comparable modules in both datasets. DistTOM similarity measure between two genes (*i* and *j*) is described as follows:

$$TOM_{ij}\;=\;\frac{l_{ij}\;+\;a_{ij}}{\min\left(k_{i},\;k_{j}\right)\;+\;1-\;a_{ij}}$$

$$DisTOM_{ij}\;=1-\;TOM_{ij}$$

where $l_{ij}=\;\underset{u}{\sum }a_{iu}a_{uj}$, $k_{i}=\;\underset{u}{\sum }a_{iu}$ is node connectivity, and *a*_*ij*_ is network adjacency.

In order to determine tissue-specificity of all modules, expression data of each module was retrieved to generate heat maps using “gplots” library of R statistical package.

### Evaluation of network robustness

Assessment of topological robustness of co-expression networks was carried out by implementing sequential node deletions on the basis of their centrality measures (Iyer et al., 2013). As a first step, topological centrality measures *viz*. degree (*k*), betweenness centrality (BC), closeness centrality (CC), and eigenvector centrality (EC), were computed to identify central nodes. Further, a certain fraction (ρ) of nodes were removed in decreasing order of centrality, with uniform random removal of nodes as a control, to examine variations in size of the largest component [σ(ρ)]. The change in σ as a function of ρ was used to determine robustness of networks in response to node deletion in the form of R-index (*R*) (Schneider et al., 2011) which was computed using following equation:

$$R=\;\frac{1}{N}\sum_{i\;=\;1}^{N}\sigma (i/N)$$

where *N* is number of nodes in network, and σ(*i*∕*N*) is fraction of nodes in the largest connected cluster. The normalization factor 1∕*N* allows comparison of robustness of networks with different sizes (Schneider et al., 2011).

During this analysis, co-expression networks were treated as unweighted in order to reduce computation complexity while determining their robustness. Additionally, vulnerability of networks to a given scheme of vertex removal was quantified by computing V-index (*V*), a value complementary to R-index (*R*), as follows:

$$V\;=\frac{1}{2}\;-R$$

Co-expression networks were compared using a composite index by combining V-indices of each of the centrality measures. The idea is to define a composite *V*_*max*_ as a vector in 5 dimensions, which was measured using following equation:

$$V_{max}=\;\sqrt{{\text{V}}_{\text{k}}{}^{2}\;+\;{\text{V}}_{\text{BC}}{}^{2}\;+\;{\text{V}}_{\text{CC}}{}^{2}\;+\;{\text{V}}_{\text{EC}}{}^{2}\;+\;{\text{V}}_{\text{R}}{}^{2}}$$

where ${{\text{V}}_{\text{k}}}^{2}$, ${{\text{V}}_{\text{BC}}}^{2}$, ${{\text{V}}_{\text{CC}}}^{2}$, ${{\text{V}}_{\text{EC}}}^{2}$, and ${{\text{V}}_{\text{R}}}^{2}$ are V-indices of centrality measures based on removal of nodes from the network in decreasing order of k, BC, CC, EC, and that from random control (*R*), respectively.

### Functional annotation and enrichment analysis of modules

Enrichment analysis of complete set of modules obtained was carried out with Singular Enrichment Analysis (SEA) algorithm of agriGO web based tool (http://bioinfo.cau.edu.cn/agriGO/) (Du et al., 2010). Significantly enriched terms were determined in all comparative conditions by comparing them against *A. thaliana* ontology as a background reference. During this procedure, Hypergeometric test with Bonferroni correction (to reduce multiple testing problems) was implemented to obtained statistically significant terms. Kyoto Encyclopedia of Genes and Genomes (KEGG) pathway analysis of significant modules were performed using a web-based program, Database for Annotation, Visualization and Integrated Discovery (DAVID) *v* 6.7 (Huang et al., 2009).

### Comparison of gene co-expression networks

To assess the module perseveration (on module-by-module basis) between these closely related species, a permutation test protocol was implemented to calculate Z-summary score using modulePreservation function in the WGCNA library (Langfelder et al., 2011). Module definitions from the reference network (*R. serpentina*) were mapped on the test network (*C. roseus*). Module preservation was also quantified in terms of significant overlap in genes, using overlapTable function (Hilliard et al., 2012), by comparing module identifiers in *R. serpentina* to match the most similar module in *C. roseus*. During this procedure, gene counts, and *p*-values (from Fisher exact test) for module assignments of these datasets were calculated. Further, on the basis of intramodular connectivity (*K*_*i*_) for each module pair, genes were compared to determine hubs that illustrate gene centrality in a given module. Genes with high *K*_*i*_ in each of the modules from reference plant, and their corresponding module from test plant, were characterized as hubs (Miller et al., 2010). For comparison of module pairs, genes with significantly high connectivity (*K*_*i*_ > 0.6) were considered. *K*_*i*_ represents normalized connectivity of gene and was computed using following equation:

$$K_{i}=\frac{k_{i}}{k_{max}}$$

where *k*_*i*_ and *k*_*max*_ is connectivity and maximal connectivity of the gene *i*, respectively.

Furthermore, shared genes from significant module pairs were also identified to get insight of differential regulatory behavior of species-specific secondary metabolite synthesis in these datasets.

In order to determine metabolites associated with significant module pairs, metabolome data of *R. serpentina* and *C. roseus* was integrated. Correlation data of these metabolomes against their corresponding transcriptome was obtained from the Plant and Microbial Metabolomics Resource (PMR; http://metnetdb.org/PMR/) using PMR metabolomic-transcriptomic co-analysis tool at a significant PCC threshold of 0.80 (Pathania and Acharya, 2016). Genes present in significant module pairs were searched against correlation data to determine module-specific metabolites for both plant species. Further, gene-metabolite networks were constructed for both plants to compare complexity in their secondary metabolism.

All computations were performed on an HPZ600 workstation and HP ProLiant DL980 G7 server running Ubuntu 12.04 and Red Hat 4.1.2 operating systems, respectively, with Intel Xeon processors.

## Results and discussion

### Identification of *Arabidopsis thaliana* homologs

**Figure 2:**
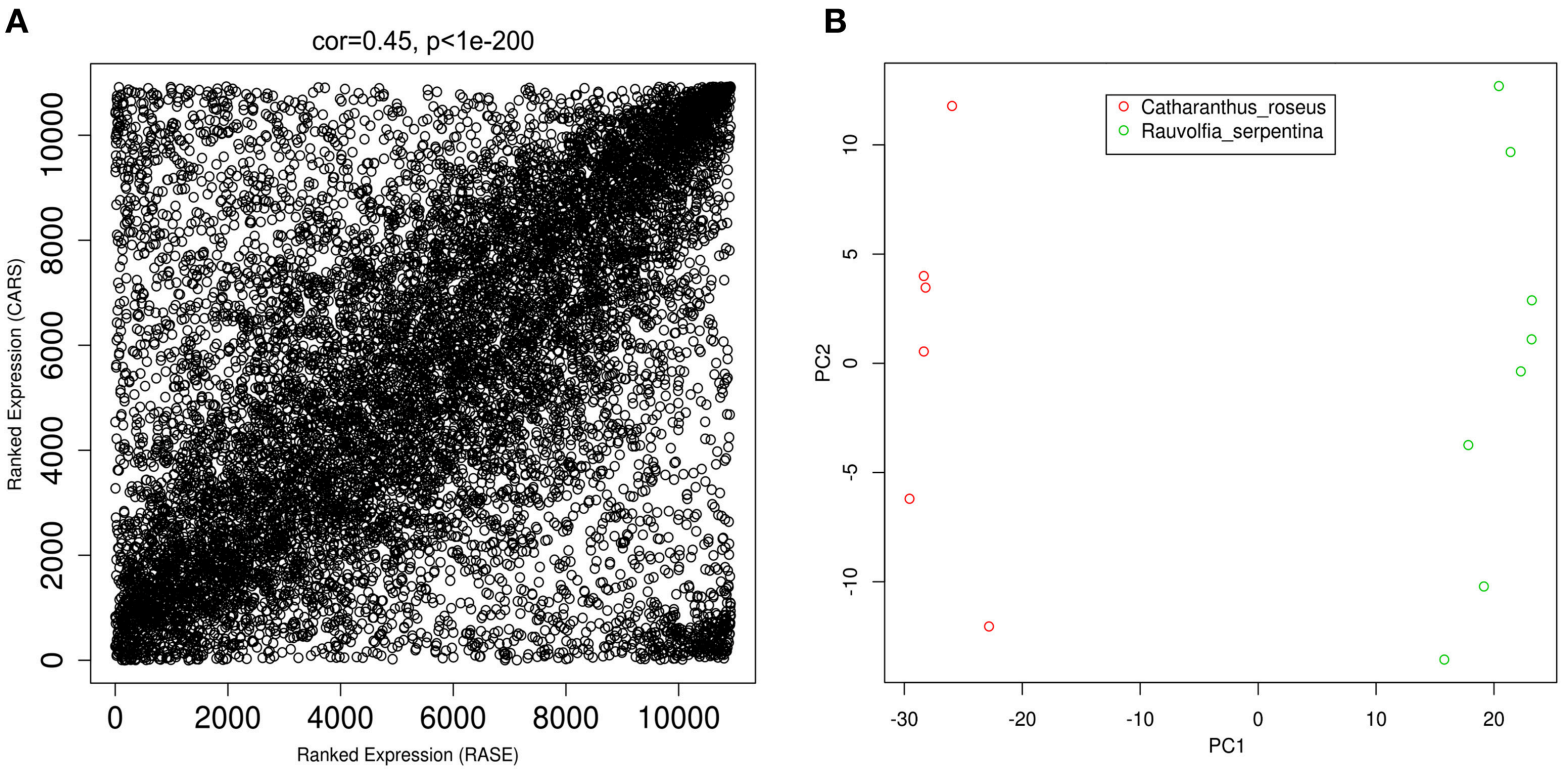
**(A)** Ranked expression correlation between two data sets samples. Each dot depicts a gene in common between data sets, where x and y axes represents ranked expression of genes in samples from *R. serpentina* and *C. roseus* datasets, respectively. **(B)** Principal component analysis (PCA) plot depict that all samples are clustered according to plant species inside the 2-dimensional space indicating clear differences in expression profiles. Red and green data points correspond to *R. serpentina* and *C. roseus* samples, respectively.

Comparative analysis often involves comparison of genes with similar identifiers. *A. thaliana* has been used as model plant to annotate various plants (Li et al., 2002) including unicellular flagellates such as *Chlamydomonas* (Merchant et al., 2007). Importantly, this plant has also been reported to annotate *R. serpentina* (Pathania and Acharya, 2016) and *C. roseus* (Schluttenhofer et al., 2014); therefore, *A. thaliana* was used to obtain similar orthologs for these plants. Annotation of complete *R. serpentina* and *C. roseus* transcripts against *Arabidopsis* proteome returned 80,829, and 67,201 significant hits with expression values for only 30,681 and 22,987 transcripts, respectively. In order to perform comparative co-expression analysis between these datasets, only the shared orthologs obtained during annotation were considered for further analysis. Network-based approach is reported to be biased toward the outliers in expression samples; therefore, it becomes necessary to perform pre-processing steps to remove such samples to ensure quality of data (Zhang et al., 2005). Also, filtering out genes that are inferred to contribute to noise has been known to facilitate meaningful inter-species comparison from gene expression in various experiments (Alter et al., 2000). In this study, filtering and pre-processing comprised of removal of 851 and 478 genes, with divergent gene expression levels, out of a total of 12,075 shared orthologs from *R. serpentina* and *C. roseus*, respectively. There was no sample outlier present in either dataset. After removal of genes, a total of 10,926 common orthologs were left for both datasets. Comparability of datasets has been defined in terms of correlation of average gene expression from different platforms (Miller et al., 2011). The high correlation with significant *p*-value (*r* = 0.45, *p* < 1e–200; Figure 2A) of average gene expression indicated comparability of *R. serpentina* and *C. roseus* datasets, facilitating subsequent analyses. Use of same RNA sequencing platform may have partly contributed to observed correlation between datasets (Miller et al., 2010).

### Identification of differentially expressed genes

Differentially expressed genes (DEGs) are primarily responsible for variations across various species that reflects in the form of differences in gene expression levels. These differences in expression level associated with the variations in regulatory mechanism which is manifested in speciation and adaptation (Romero et al., 2012). Since such DEGs have been reported to be involved in secondary metabolite synthesis (Tao et al., 2013; Zhou et al., 2015), they are expected to be responsible for differential metabolite synthesis in *R. serpentina* and *C. roseus*. Microarray data has extensively been used to identify DEGs among various datasets (Wu et al., 2014), but recently increased use of RNA sequencing has emerged out to be an eminent alternative to carry out differential expression studies (Chen et al., 2011). Since expression data analysis identifies genes whose expression pattern changes under variation of phenotype and different experimental conditions, such genes with differences in expression may be contribute to diversification of downstream of TIA pathway that led to species-specific synthesis of alkaloids. Moreover, these DEGs have been found to responsible for diversity in secondary metabolite synthesis; hence, identification of such genes may help in exploration of complexity of biosynthetic mechanisms (Shaik and Ramakrishna, 2013; Liang et al., 2015). To characterize such genes responsible for variation in species-specific metabolite synthesis of *R. serpentina* and *C. roseus*, differences in expression profiles were analyzed. A total of 4647 transcripts were left at variance threshold from a total of 10,926 common *Arabidopsis* orthologs. Statistical significance of these transcripts was further verified by implementing *t*-test which resulted in identification of 858 genes to be differentially expressed (*q* ≤ 0.05). Multiple testing corrections were performed to reduce the false positives during statistical validation. PCA was performed using all DEGs that reduces dimensionality of multivariate data while preserving most of variance for easy data perception and visualization (Varmuza and Filzmoser, 2009). A clear separation in both of the datasets was observed without any mixing which indicates their unique distinguishable expression profiles (Figure 2B).

### Characterization of co-expression modules

Co-expression networks are generated on the basis of correlation patterns among various samples which facilitates the characterization of candidate genes involved in important biological processes (Oldham et al., 2006; Miller et al., 2010). In this study, weighted co-expression networks were generated using expression profiles data of different tissues from *R. serpentina* (8) and *C. roseus* (6). Earlier studies support sufficiency of the number of samples for this expression data to identify new candidate genes using co-expression analysis (Góngora-Castillo et al., 2012; Paul et al., 2014; Schluttenhofer et al., 2014; Pathania and Acharya, 2016). Co-expression networks obtained for both plant species were signed networks that allowed network to retain positively as well as negatively correlated genes in various sets which correspond to up- and down-regulated genes, respectively. Network topologies were evaluated using WGCNA and the resulting networks, with an appropriate value of soft-threshold (β = 14), were found to have approximate scale-free topology and presence of modularilty. The scale free topology revealed the robustness of both networks against random disruptions since such networks are most likely to hit a node with only a few neighbors, and thereby minimally affecting their integrity (Emmert-streib and Dehmer, 2008). Presence of modular structure enables characterization of functionally important genes that work in coordinated manner (Porter et al., 2009). These modules correspond to functional units of genes that work in an integrative manner; therefore, identification of such modules is a step toward understanding complexity of biological processes (Huss and Holme, 2007). Clusters in co-expression networks of both datasets were obtained to simplify their comparative analysis as well as to identify their tissue-specific expression. Similarly, identification of such modules was performed through autonomous clustering of network, i.e., without any prior information. TOM is a biologically relevant and robust method that works on the basis of high-order neighborhoods in order to measure pairwise interconnectedness among genes (Yip and Horvath, 2007). It also reduces noise and irrelevant interactions to compute dissimilarity TOM (disTOM) (Horvath, 2011). Hierarchical clustering was implemented on disTOM to obtain modules with each of them have similar gene expression profiles. A total of 8 color coded modules (black, blue, brown, green, gray, red, turquoise, and yellow) were determined by dendrogram cutting (Langfelder et al., 2008). During this process, a “deepsplit” threshold of 0 and 2 were considered for *R. serpentina* (Figure S1A) and *C. roseus* (Figure S1B), respectively. These values were arrived at after visual inspection of dendrogram plots to have comparable number of modules. These modules had size range of 51–302, and 7–214 in *R. serpentina* and *C. roseus*, respectively. It is important to note that modules were assigned a color according to size gradient within a network. Genes of gray colored module do not cluster into any other module since they have too dissimilar expression pattern to form a cluster. A modular organization of genes which tended to form clusters (based on their high correlation) could be easily anticipated from gene dendrograms.

### Robustness of modules

**Figure 3:**
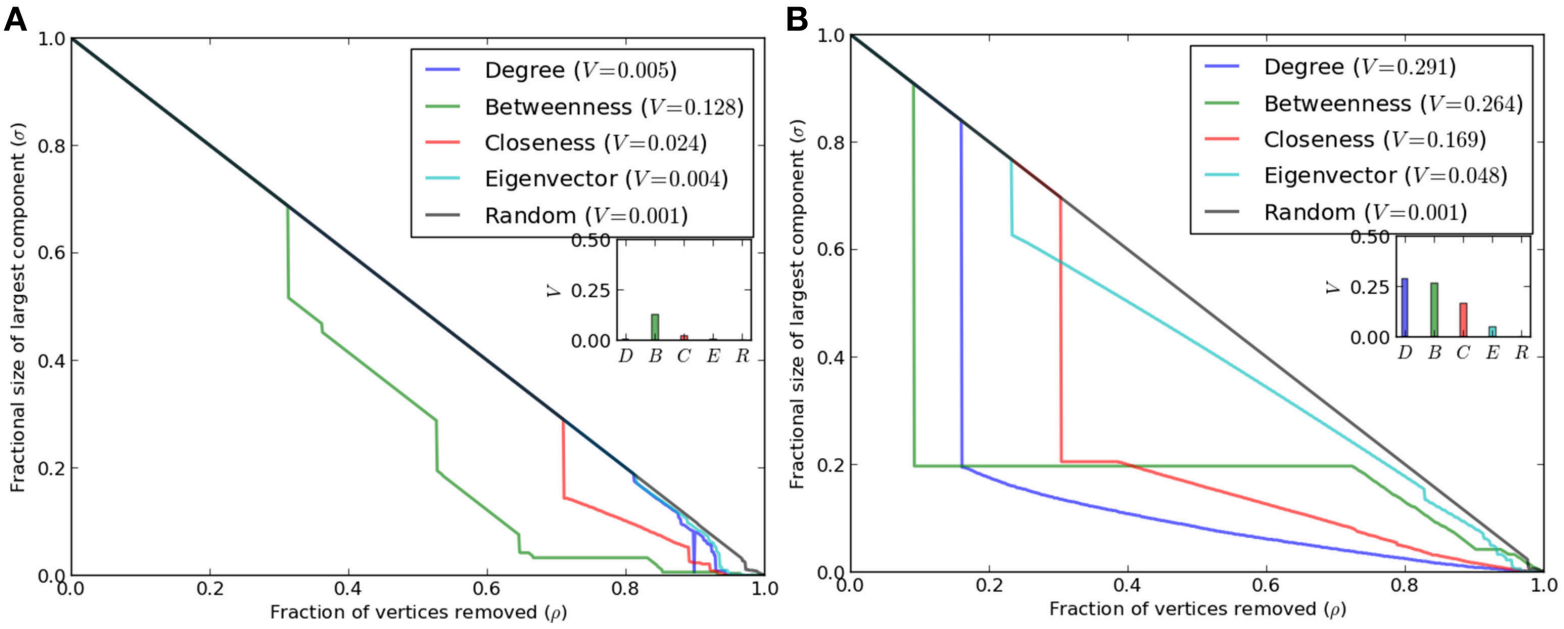
**Analysis of topological robustness of the both networks (A) *R. serpentina* and (B) *C. roseus* via plotting of a sequential node deletion against changes in the size of the largest component, σ(ρ), when the fraction ρ of the vertices (nodes) was removed**. The results indicate the high network robustness of *R. serpentina* as compared to *C. roseus*.

Robustness of complex systems against fragmentation under failures is a critical feature, since removal of key nodes affects overall functionality and impairs performance of networked systems (Iyer et al., 2013). Robustness has been studied for various empirical networks with the help of topological metrics by random or systematic deletion of nodes. Although scale-free networks are robust toward random deletions, they are vulnerable to targeted attacks against hub nodes. Reduction in size of the largest component of network (due to removal of hub nodes) may lead to loss of interactions between many nodes. Because these key nodes maintain network integrity, deletion of such nodes may further affect various biological processes. Figure 3 depicts relevance of various parameters toward structural integrity of network, measured in terms of the size of largest component. The impact of deletion of nodes ranked according to a parameter was compared with that of random deletion. While there is difference in extent of relevance among parameters, the co-expression network of *R. serpentina* emerges as extremely robust even under targeted attack (Figure 3A). Whereas, co-expression network of *C. roseus* is fragile and breaks down easily under targeted attack (Figure 3B). For *C. roseus*, a sudden decline in the network integrity was observed at ~30% deletion of ranked nodes for all given centrality measures. Afterwards graph showed a gradual decrease in network breakdown. On the contrary, a gradual decrease in the size of network was observed in *R. serpentina* i.e., size of largest connected component was kept at ~65% of node deletion. This result is indicative of network robustness of *R. serpentina*. Similarly, *V*−*index*_*max*_ complements above results with values of 0.13 and 0.43 for *R. serpentina* and *C. roseus*, respectively, indicating a higher robustness of *R. serpentina*. Evolutionarily speaking robust networks are expected to evolve from relatively primitive topologies with lesser robustness (Ciliberti et al., 2007). Hence we infer that mechanisms of secondary metabolite synthesis in *R. serpentina* are more complex and may have evolved over *C. roseus* under environmental stresses. While when compared on a feature such as pollen structure *R. serpentina* is primitive to *C. roseus* under evolutionary scale, mechanisms of root indole alkaloids synthesis in *R. serpentina* are reported to be highly complex and integrated to hormonal- or elicitor-mediated signaling pathways under plant-pathogen response (Pathania and Acharya, 2016). In contrast, major TIAs in *C. roseus* are known to be derived from aerial part and are less affected against plant-pathogen interaction (De Luca and St Pierre, 2000; Zhu et al., 2015). In summary, secondary metabolite synthesis from TIA pathway is comparatively more complex in *R. serpentina* which is supported by robustness analysis of co-expression networks (Figure 3).

### Preservation analysis of signature pathways

**Figure 4:**
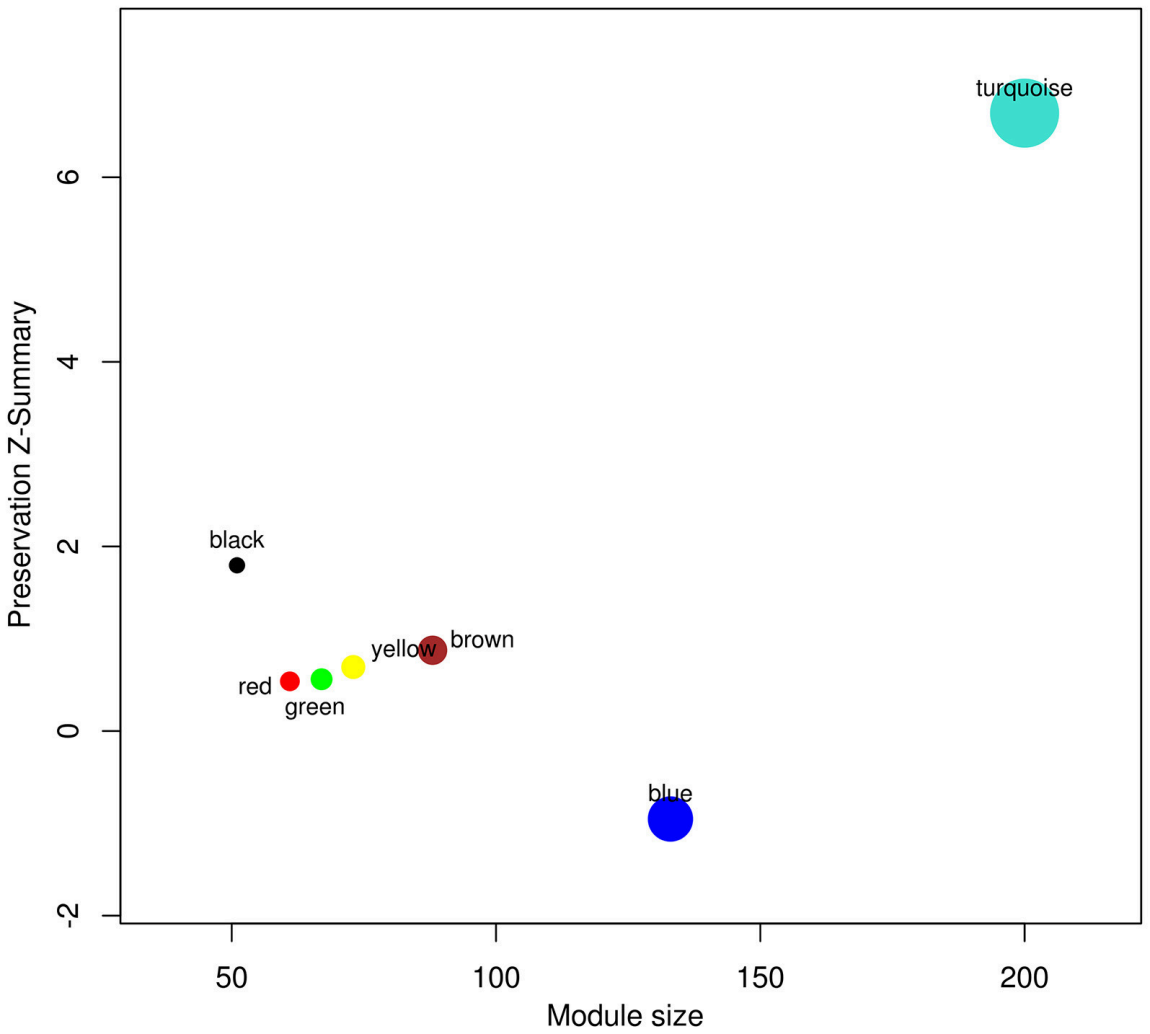
**The Z-summary statistic (y-axis) of the reference (*R. serpentina*) data modules against test (*C. roseus*) network modules is plotted as a function of module size**. Each circle represents a module labeled by a color and module name.

**Figure 5:**
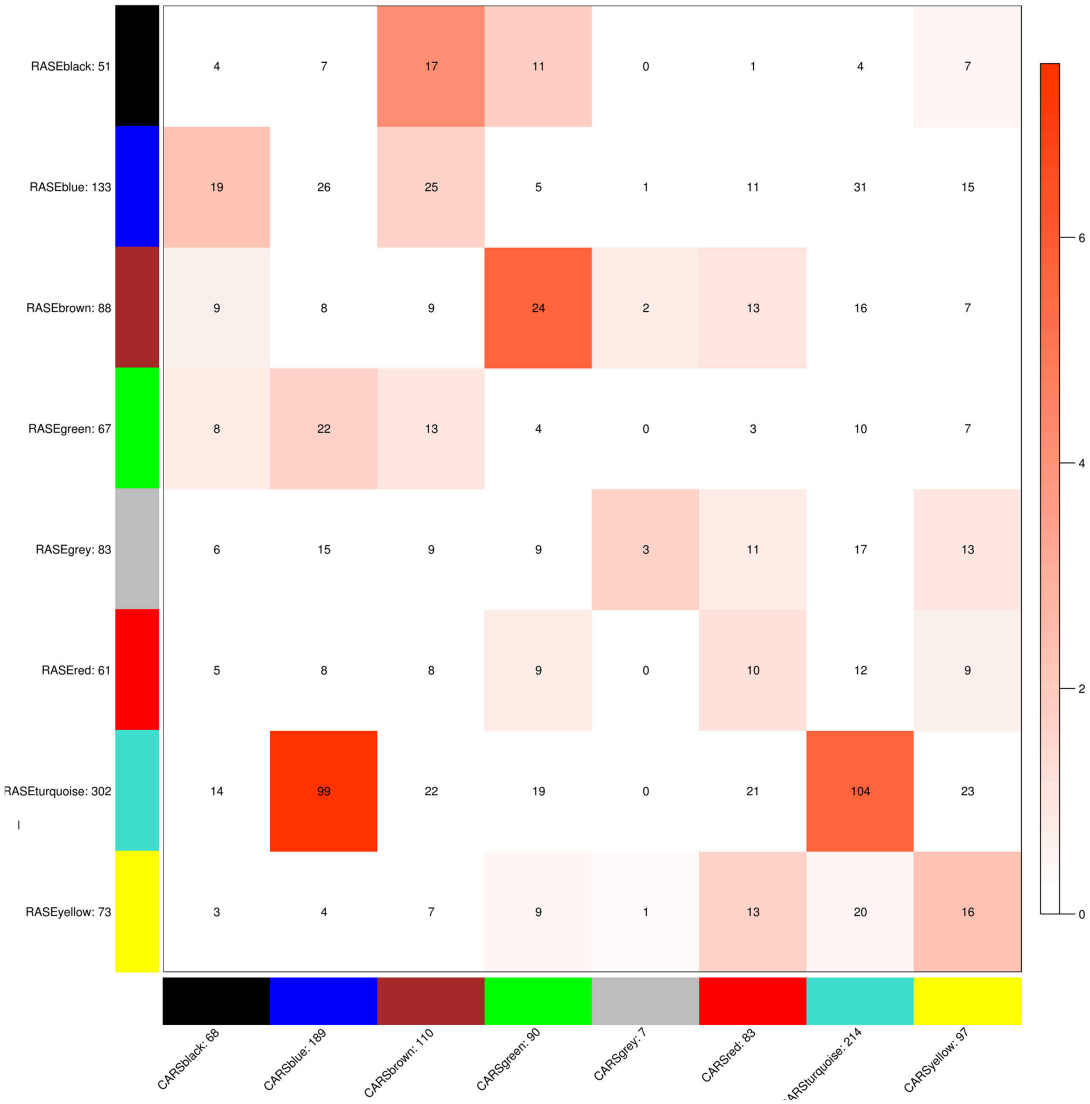
**Module preservation plot representing the number of genes conserved between the modules of test network (*C. roseus*) and reference network (*R. serpentina*)**. Preservation significances are represented by the depth of red color.

Signature pathways are reported to be conserved across species, and molecular identifiers from the model plant can be used to determine such core pathways in non-model plants (Wang et al., 2013; Pathania and Acharya, 2016). WGCNA was implemented to obtain preservation statistics which is a composite strategy to measure Z-summary score on the basis of network density and connectivity of modules. It characterizes the functional connectivity between networks of these two species based on given hypothesis that modules identified in reference network also persist in test network. Modules with Z-summary score less than two are known to be diverse and have no evidence of conservation, whereas Z-summary score between 2 and 10 and greater than 10 corresponds to adequately and strongly preserved modules, respectively (Langfelder et al., 2011). Z-summary score also depends on module size, i.e., conservation of higher number of nodes is more significant as compared to module with fewer nodes. While comparing modules from test network (*C. roseus*) against reference network (*R. serpentina*), Z-summary score of modules preservation were in range from –0.95 to 6.69 (Table 1, Figure 4). Only one module (turquoise) was found to be conserved with Z-summary score of 6.69 implying probable core mechanism in both plants. All other modules had Z-summary scores < 2. Interestingly, a total of three modules, including turquoise module, were found to share significant number of overlapped genes (Figure 5). This indicated that preservation criterion also depends upon the number of genes common between module pair from both networks. A total of three modules including turquoise, black, and brown modules from *R. serpentina* were significantly conserved with turquoise as well as with blue, brown, and green modules of *C. roseus*, respectively. Inconsistent module preservation between these networks revealed dissimilarity in synthesis of metabolites which is also reflected by difference in tissues-specific synthesis of major indole alkaloids in *R. serpentina* (Lelek and Furedi Szabo, 1961; Nammi et al., 2005; Jerie, 2007; Dey and De, 2011) and *C. roseus* (Mishra and Kumar, 2000; Shukla et al., 2006). The turquoise module from *R. serpentina* network shared 104 and 99 genes (statistically significant) against turquoise and blue modules of *C. roseus*, respectively. This significant overlap of genes strongly complemented by high preservation score highlights functional importance of turquoise module in Apocynaceae family and may represents a signature pathway (Langfelder et al., 2011).

**Table 1:** **Table represents the preservation statistics of modules from reference (*R. serpentina*) and test (*C. roseus*) networks**.

**S. No**.	**Modules**	**Module size**	**Z_summary_ score**	**Correlation of kME values (All-module genes)**	**Correlation of kME values (in-module genes)**
1	Turquoise	200	6.6945826	[0.36, 1.2e–27]	[0.4, 5e–13]
2	Gold	50	2.8849298	–	–
3	Black	51	1.7958002	[0.18, 1.1e–07]	[0.11, 0.44]
4	Gray	83	1.4699666	[0.23, 9.2e–12]	[−0.29, 0.0078]
5	Brown	88	0.8751528	[0.28, 6.4e–17]	[−0.13, 0.23]
6	Yellow	73	0.6934353	[0.28, 6.4e–17]	[−0.079, 0.51]
7	Green	67	0.5611602	[0.24, 1e–12]	[0.24, 0.05]
8	Red	61	0.5386991	[0.34, 1.2e–24]	[−0.11, 0.4]
9	Blue	133	−0.9531561	[−0.016, 0.64]	[−0.029, 0.74]

Module membership (kME) of the genes from each module were compared and computed for their correlation. Modules which are having a high correlation with significant p-value are highly preserved. Where, all- and in-module genes include the correlation of all genes in module and subset of genes, respectively, to display hub-gene conservation.

**Figure 6:**
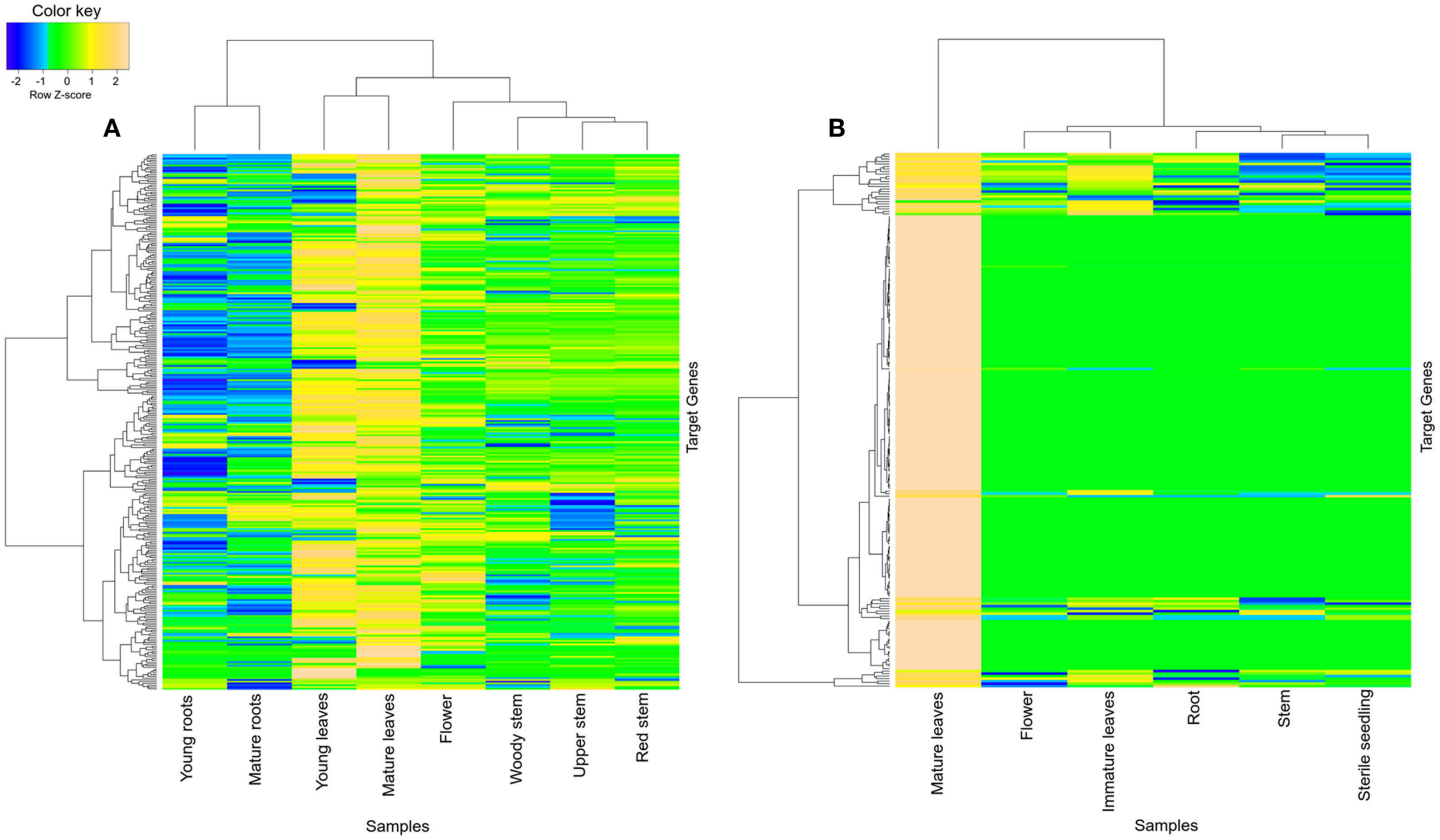
**Heatmap of transcripts using expression data of different tissues**. Heatmap is depicting tissue-specific expression of transcripts of turquoise modules in **(A)** young as well as mature leaves and **(B)** mature leaves of *R. serpentina* and *C. roseus*, respectively, where average expression is calculated based on normalized transcriptomics data. The “gplots” library of R statistical package is used to plot heatmap.

Turquoise module was found to share various significantly enriched GO terms between datasets (Figures S2, S3): *response to stimulus* (GO:0050896), *response to abiotic stimulus* (GO:0009628), *response to chemical stimulus* (GO:0042221), *response to inorganic substance* (GO:0010035), *response to temperature stimulus* (GO:0009226), *response to water deprivation* (GO:0009414), *response to stress* (GO:0006950), *photosynthesis* (GO:0015979), *photosynthesis, light reaction* (GO:0019684), *oxidation reduction* (GO:0055114), *metabolic process* (GO:0008152), *generation of precursor metabolites* (GO:0006091), and *cellular nitrogen compound metabolic process* (GO:0034641). Turquoise module was also found to have high tissue-specific expression in leaves of *R. serpentina* (both young as well as mature leaves) (Figure 6A) and *C. roseus* (only in mature leaves) (Figure 6B). Tissue-specific expression of these modules in leaves emphasizes their involvement in photosynthesis dependent synthesis of precursors and its regulation in both datasets. Similarly, turquoise module of *R. serpentina* was characterized with few common enriched terms with blue module of *C. roseus* (Figure S4): *chlorophyll metabolic process* (GO:0015994), *porphyrin metabolic process* (GO:0006778), *tetrapyrrole metabolic process* (GO:0033013), and *pigment biosynthetic process* (GO:0046148). The blue module was also represented with tissue-specific expression in both immature and mature leaves (Figure S5). These shared GO terms signify similarity of primary metabolite synthesis as well as upstream of TIA pathway in *R. serpentina* and *C. roseus*. Both of these plants have been reported to share initial steps of TIA pathway which are localized in leaves (O'Connor and Maresh, 2006; Guirimand et al., 2010); this result complements our results. Similarly, pathway enrichment analysis of *R. serpentina* turquoise module (Figure S6A, Table S1) presented top two (photosynthesis and biosynthesis of plant hormones) and one (biosynthesis of plant hormones) hits common with turquoise (Figure S6B, Table S2) and blue (Figure S6C, Table S3) module of *C. roseus*, respectively. Our findings from pathway analysis were complemented by agriGO enrichment results. Plant hormones have been reported to act as secondary messengers to induce expression of enzymes required for the synthesis of precursors for TIA pathway (Menke et al., 1999). Pathway analysis revealed the initiation of TIA pathway under the hormone-/elicitor-induced signaling in both plants. Conservation of photosynthesis process and upstream of TIA pathway highlights their significance as signature pathway of Apocynaceae family.

**Figure 7:**
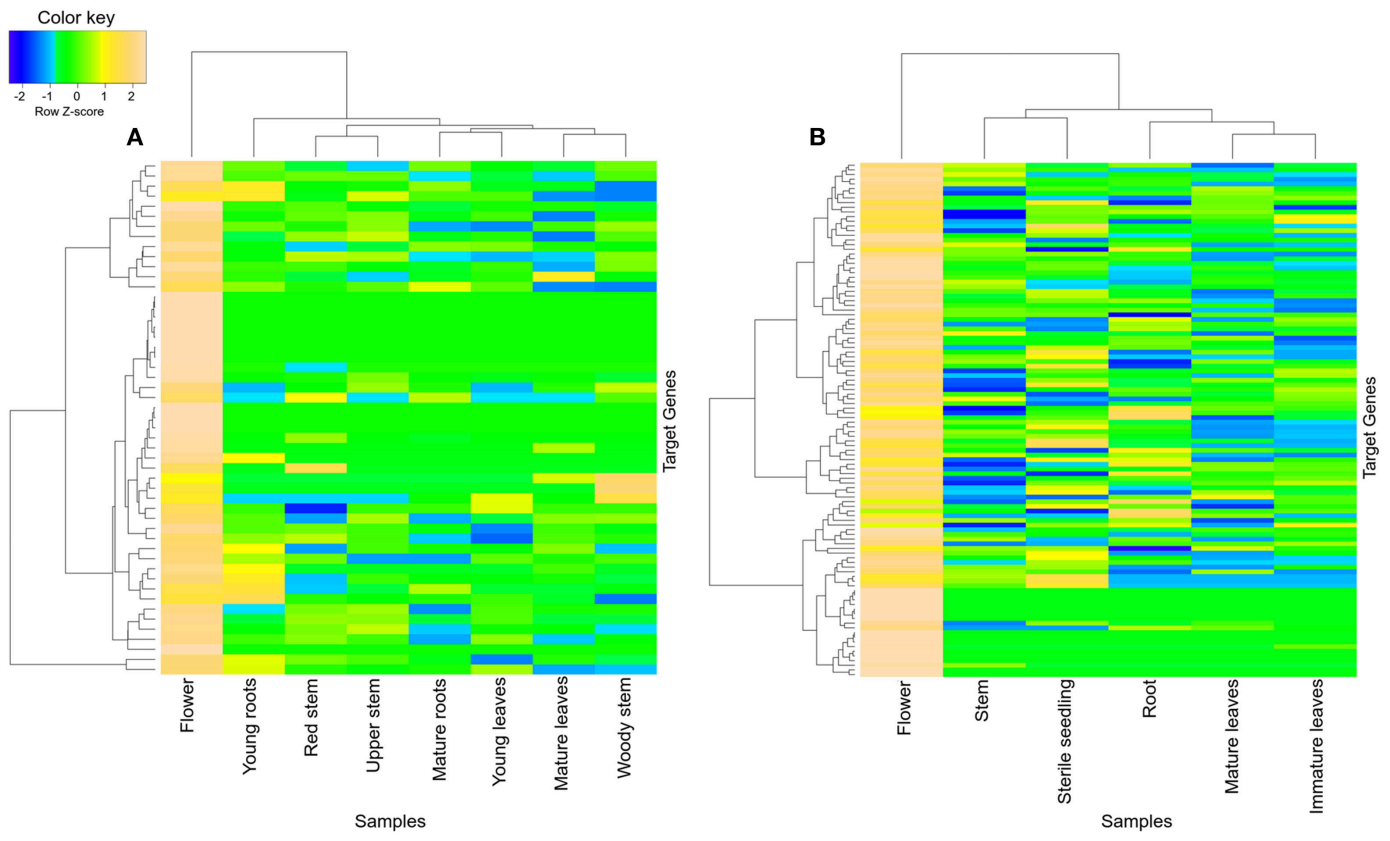
**Heatmap of transcripts using expression data of different tissues**. Heatmap is depicting tissue-specific expression of both transcripts of **(A)** black and **(B)** brown modules in flower, from *R. serpentina* and *C. roseus*, respectively, where average expression is calculated based on normalized transcriptomics data. The “gplots” library of R statistical package is used to plot heatmap.

Similarly, black module of the *R. serpentina* was found to have significant overlap of genes (>30%) with brown module of *C. roseus* (Figure 5), and both of these modules presented with higher tissue-specific expression in flower (Figure 7). The significant GO terms which were shared by both these modules (Figures S7, S8) are as follows: *response to stimulus* (GO:0050896), *biological regulation* (GO:0065007), *localization* (GO:0051179), *establishment of localization* (GO:0051234), *transport* (GO:0006810), *macromolecule metabolism process* (GO:0043170), *biosynthetic process* (GO:0009058), *protein metabolic process* (GO:0019538), *cellular macromolecule metabolic process* (GO:0044260), and *cellular biosynthetic process* (GO:0044249). In addition, the GO enrichment analysis also revealed important enriched terms such as macromolecular and protein biosynthetic processes, transport etc., indicative of preferential shift of active metabolism to cellular proliferation activities which are further involved in plant reproductive systems (Balbuena et al., 2012). These biological processes are related to stigma development and its fusion with pollen (Nazemof et al., 2014). This is further complemented by tissue-specific expression of modules in flower (Figure 7). Also, pathway analysis of this module pair presented significant shared hits such as biosynthesis of phenylpropanoids, flavone and flavonol biosynthesis, phenylpropanoid biosynthesis, and biosynthesis of alkaloids derived from shikimate pathway (Figure S9, Tables S4, S5). This observation is complemented by high expression of genes from this module pair in flower tissue (Vogt, 2010). These results indicated that these closely related plants share similar core process of stigma development, mode of reproduction, and flavonoid synthesis.

### Diversity in major alkaloid synthesis

**Figure 8:**
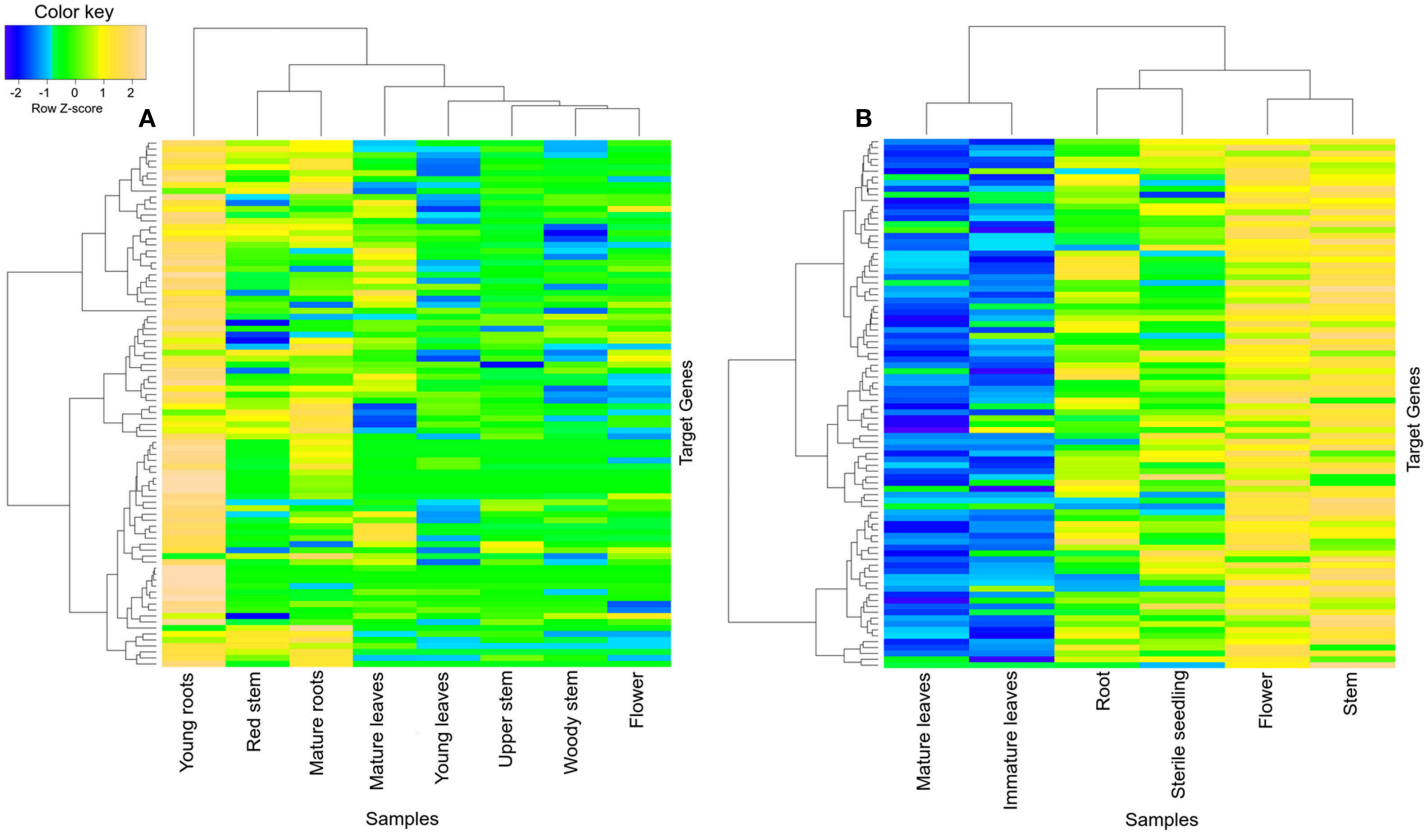
**Heatmap of transcripts using expression data of different tissues**. Heatmap is depicting tissue-specific expression of transcripts of **(A)** brown module in young roots and **(B)** green module in aerial part (flower and stem), from *R. serpentina* and *C. roseus*, respectively, where average expression is calculated based on normalized transcriptomics data. The “gplots” library of R statistical package is used to plot heatmap.

The brown module from *R. serpentina* network was found to share significant number of genes (~27%) with green module of *C. roseus* (Figure 5). Despite of having significant number of shared genes, only one common GO term (response to chemical stimulus, GO:0042221) was observed (Figures S10, S11). This pair also presented with different tissue-specific expression, mainly in young roots (Figure 8A) and aerial parts (Figure 8B) of *R. serpentina* and *C. roseus*, respectively. Majority of important metabolites are reported to be synthesized from roots (O'Connor and Maresh, 2006; Pathania and Acharya, 2016) and aerial part (Zhong et al., 2010; Zhu et al., 2015) in *R. serpentina* and *C. roseus*, respectively. Thus, despite sharing of genes, the site of expression was different for both plants pointing to their involvement in species-specific synthesis of major indole alkaloids. This difference in expression behavior may have led to bifurcation of synthesis of these alkaloids and further diversification of these two closely related plants under evolutionary time scale.

**Figure 9:**
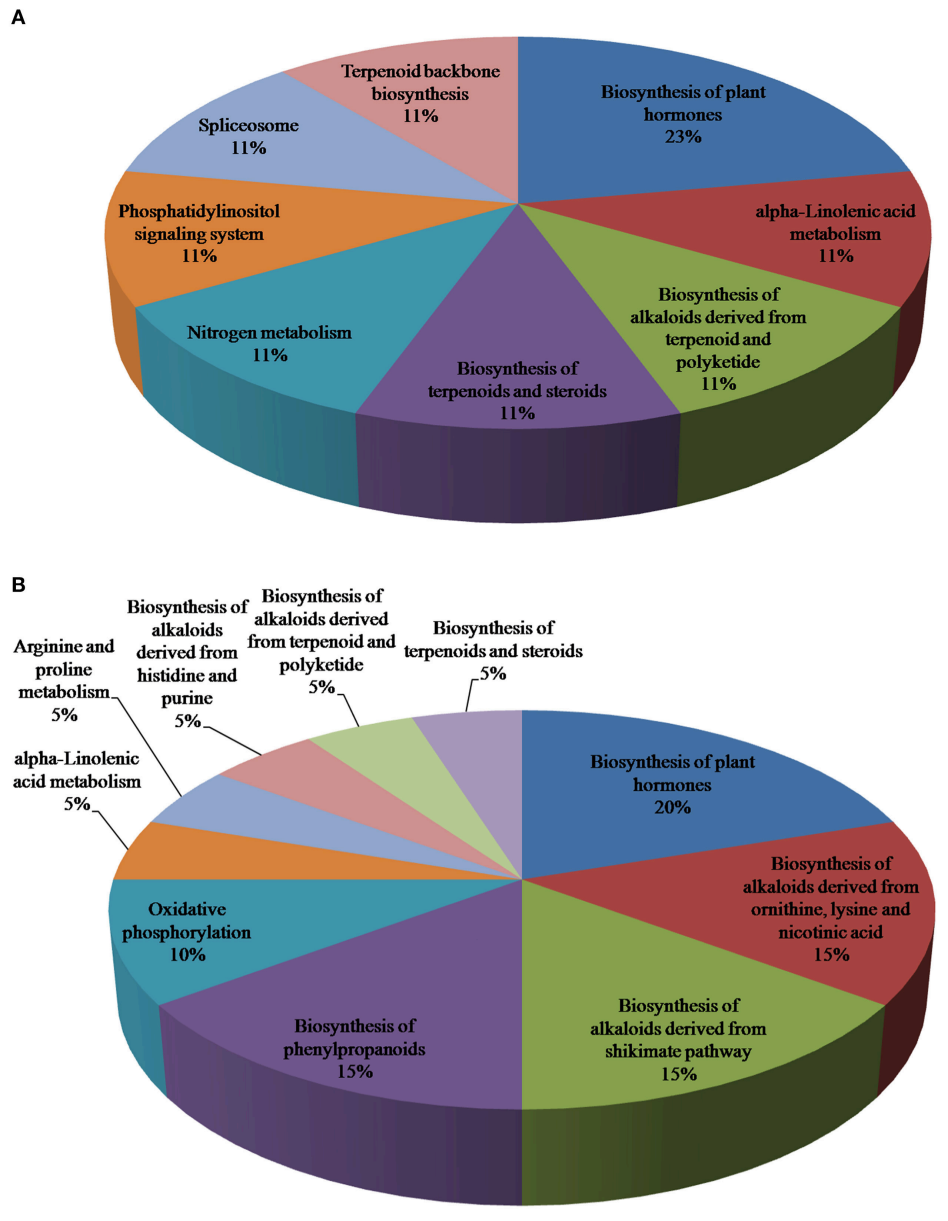
**Pie chart representing the count of significant KEGG pathways of (A) brown and (B) green modules from *R. serpentina* and *C. roseus*, respectively, using DAVID**.

The brown module in *R. serpentina* network was enriched with following terms (Figure S10): *response to stimulus* (GO:0050896), *response to abiotic stimulus* (GO:0009628), *response to chemical stimulus* (GO:0042221), *response to inorganic substance* (GO:0010035), *response to organic substance* (GO:0010033), *response to stress* (GO:0006950), *defense response* (GO:0006952), *response to carbohydrate stimulus* (GO:0009743), *response to chitin* (GO:0010200), *biological regulation* (GO:0065007), *regulation of biological process* (GO:0050789), *regulation of cellular process* (GO:0050794), *regulation of metabolic process* (GO:0019222), *nitrogen compound metabolic process* (GO:0006807), *regulation of nitrogen compound metabolic process* (GO:0051171), *regulation of cellular metabolic process* (GO:0031323), *regulation of primary metabolic process* (GO:0080090), *regulation of nucleobase, nucleoside, nucleotide, and nucleic acid metabolic process* (GO:0019219), *nucleobase, nucleoside, nucleotide, and nucleic acid metabolic process* (GO:0006139), *regulation of biosynthetic process* (GO:0009889), *biosynthetic process* (GO:0009058), *cellular biosynthetic process* (GO:0044249), *regulation of cellular biosynthetic process* (GO:0031326), *regulation of macromolecule metabolic process* (GO:0060255), *regulation of transcription* (GO:0045449), *RNA metabolic process* (GO:0016070), *regulation of macromolecule biosynthetic process* (GO:0010556), *macromolecule metabolic process* (GO:0043170), *cellular macromolecule metabolic process* (GO:0044260), *transcription* (GO:0006350), *cellular macromolecule biosynthetic process* (GO:0034645), *macromolecule biosynthetic process* (GO:0009059), *regulation of gene expression* (GO:0010468), *protein metabolic process* (GO:0019538), *cellular protein metabolic process* (GO:0044267), *gene expression* (GO:0010467), *macromolecule modification* (GO:0043412), *protein modification process* (GO:0006464), and *post-translational protein modification* (GO:0043687). These enriched terms have been reported to be associated with synthesis of specialized secondary metabolites under various stimuli (Weng and Noel, 2012; Pathania and Acharya, 2016). Comparison of top five pathways between this module pair revealed presence of only one common pathway i.e., biosynthesis of plant hormones (Figure 9). Interestingly, two pathways including spliceosome and terpenoid backbone biosynthesis (from brown module of *R. serpentina*) (Figure 9A, Table S6) were not present in corresponding green module (Figure 9B, Table S7). These pathways are known to be associated with disease resistance and TIAs synthesis, respectively (Zhang et al., 2013), augmented by expression of brown module in roots. Similarly, pathway analysis of green module was found to be associated mainly with phenylpropanoid synthesis. Majority of the pathways (biosynthesis of phenylpropanoids, phenylalanine metabolism, phenylpropanoid biosynthesis, and tryptophan metabolism) were observed to be associated with phenylpropanoid synthesis (Figure 9B) (Yao et al., 1995; Fraser and Chapple, 2011), complemented by expression of green module in flower.

Spliceosome and terpenoid backbone biosynthesis were two significant pathways identified from pathway enrichment study (which was complemented by agriGO enrichment analysis) that are possibly involved in synthesis of major indole alkaloids under various stresses in *R. serpentina* in contrast to phenylpropanoid synthesis in *C. roseus*. These indole alkaloids are reported to be synthesized in roots of *R. serpentina* and flowers of *C. roseus*, which is further complemented by tissue-specific expression of these modules (Figure 8). These findings indicate that the late steps of TIA pathway may be associated with brown module of *R. serpentina* and have evolved over *C. roseus* to synthesize species-specific metabolites.

### Divergent hub genes

Despite of significant sharing (Figure 5), brown-green pair had different tissue-specific expression of all three conserved module pairs which could be possibly explained by differences in regulatory mechanisms (Reece-Hoyes et al., 2013). Presence of only one common GO term in this module pair complemented their difference in tissue-specific expression. We hypothesized that this could be due to differences in central genes of these modules and thereby, variations in regulatory mechanisms to synthesize metabolites. The hubs (central genes) are known to be more essential for survival and cellular growth rate of organisms (Batada et al., 2006). Knowing that gene connectivity plays important roles in plant phenotypes (Weston et al., 2008), we studied difference in properties of central genes and their connection to phenotypic variations.

Brown module from *R. serpentina* had 17 shared genes with green module of *C. roseus* (Table 2) at the threshold of *K*_*i*_≥0.6, out of which three genes (AT2G23290.1, AT1G72520.1, and AT2G01300.1) had significant difference in connectivity. These genes had higher intramodular connectivity (*K*_*i*_) in *C. roseus* as compared to *R. serpentina* (Table 2). The transcript identifier AT2G23290 was annotated as MYB70, a member of R2R3 MYB TF family genes which are known to be involved in phenylpropanoid synthesis (Borevitz, 2000), anthocyanin production (Paz-Ares et al., 1987; Goff et al., 1992; Ramsay and Glover, 2005), and reproductive organ development in flowers under stress (Boavida et al., 2011). AT1G72520 (*Arabidopsis thaliana* lipoxygenase 4; ATLOX4) is reported to be involved in stamen and petal development (Caldelari et al., 2011). AT2G01300 is known to be involved in pollination and floral organ development (Hennig et al., 2004; Zahn et al., 2005). These three genes, with most significant difference in their connectivity between these plants, emerged as the central to green module *C. roseus*, and are known to be involved in reproductive system which is further complemented by gene expression of this module in flower. On the contrary, these three genes have significantly lesser value of connectivity in corresponding brown module of *R. serpentina* which possibly alludes to their down regulation in major root indole alkaloids synthesis. Difference in *K*_*i*_ of these genes (possibly due to rewiring of their interactions) may be responsible for differences in tissue-specific as well as species-specific metabolite synthesis between these medicinal plants as evident from data in Figure 8. MYB70, the only TF identified among these genes, could be critical in differential regulatory mechanisms.

**Table 2:** **Intramodular connectivity (*K*_*i*_) of shared genes from brown and green module of *R. serpentina* (RASE) and *C. roseus* (CARS), respectively**.

**S. No**.	**ATIDs**	**RSIDs**	**K_i_ of RASE**	**CRIDs**	**K_i_ of CARS**
1	AT2G33170.1	rsa_locus_20040_iso_1_len_628_ver_2	1	cra_locus_2641_iso_1_len_3992_ver_3	0.983
2	AT2G23755.1	rsa_locus_60320_iso_1_len_335_ver_2	0.996	cra_locus_34353_iso_1_len_643_ver_3	0.935
3	AT1G58420.1	rsa_locus_17189_iso_1_len_765_ver_2	0.977	cra_locus_10634_iso_1_len_763_ver_3	0.939
4	AT1G11050.1	rsa_locus_318_iso_2_len_3548_ver_2	0.919	cra_locus_2311_iso_1_len_4155_ver_3	0.906
5	AT4G22600.1	rsa_locus_21779_iso_1_len_1439_ver_2	0.917	cra_locus_18418_iso_1_len_1428_ver_3	0.906
6	AT2G15760.1	rsa_locus_1097_iso_3_len_975_ver_2	0.888	cra_locus_893_iso_1_len_1250_ver_3	0.811
7	AT3G07490.1	rsa_locus_13412_iso_1_len_521_ver_2	0.877	cra_locus_1192_iso_1_len_1528_ver_3	0.876
8	AT5G01830.1	rsa_locus_22614_iso_1_len_2313_ver_2	0.874	cra_locus_2457_iso_1_len_2491_ver_3	0.793
9	AT2G40000.1	rsa_locus_54630_iso_1_len_297_ver_2	0.852	cra_locus_566_iso_2_len_1446_ver_3	0.966
10	AT5G06710.1	rsa_locus_8304_iso_1_len_640_ver_2	0.828	cra_locus_16080_iso_1_len_1780_ver_3	0.727
11	AT1G76350.1	rsa_locus_6660_iso_2_len_1669_ver_2	0.819	cra_locus_1842_iso_1_len_1728_ver_3	0.952
12	AT3G57630.1	rsa_locus_13327_iso_2_len_2547_ver_2	0.785	cra_locus_3747_iso_1_len_3248_ver_3	0.929
13	AT5G67300.1	rsa_locus_1417_iso_2_len_1458_ver_2	0.735	cra_locus_94_iso_1_len_1200_ver_3	0.872
**14**	**AT2G23290.1**	**rsa_locus_4970_iso_2_len_1415_ver_2**	**0.663**	**cra_locus_94_iso_3_len_769_ver_3**	**0.910**
**15**	**AT1G72520.1**	**rsa_locus_32_iso_6_len_1369_ver_2**	**0.653**	**cra_locus_842_iso_2_len_1157_ver_3**	**0.945**
**16**	**AT2G01300.1**	**rsa_locus_8505_iso_1_len_753_ver_2**	**0.622**	**cra_locus_10412_iso_1_len_790_ver_3**	**0.935**
17	AT5G61510.1	rsa_locus_10177_iso_1_len_1131_ver_2	0.615	cra_locus_4109_iso_1_len_1183_ver_3	0.804

Genes with significant difference in K_i_ are bold.

Despite of shared genes between this conserved module pair (brown-green) (Figure 5), these modules show tissue-specific expression (Figure 8). Therefore, metabolites synthesized by these modules were determined by integrating the metabolomics data of both plant species, and were further compared for the complexity in secondary metabolism. A total of 28 and 4 transcripts from brown and green modules of *R. serpentina* and *C. roseus*, respectively, were found to have significant correlation with at least one metabolite reported in PMR. Out of 17 shared genes (Table 2), 6 and 2 transcripts of *R. serpentina* and *C. roseus*, respectively, were found to correlate with corresponding metabolites. All these six transcripts in *R. serpentina* were found to be involved in the synthesis of ajmaline/sarpagine-type alkaloids of the late steps of TIA pathway (O'Connor and Maresh, 2006). Moreover, one of the reported candidate genes AT2G01300.1 (rsa_locus_8505_iso_1_len_753_ver_2) in *R. serpentina* was found to be associated with the synthesis of major root indole alkaloids such as ajmalicine, reserpine, ajmalicine, and serpentine (O'Connor and Maresh, 2006) that complements the tissue-specific expression of this brown module in roots. The annotation for this locus is incomplete for its biological process and molecular function, and is reported to be expressed in roots in addition to leaves and flowers. Similarly, two transcripts of *C. roseus* (AT2G23290.1, cra_locus_94_iso_3_len_769_ver_3 and AT1G72520.1, cra_locus_842_iso_2_len_1157_ver_3) were found to be associated with the synthesis of metabolites (such as tabersonine, ajmalicine, and akuammicine) known to be produced in aerial parts (Mukhopadhyay and Cordell, 1981; Iwase et al., 2005; Murata and De Luca, 2005). All these observations highlighted the accuracy of protocol implemented as well as significance of the results.

Comparison of gene-metabolite networks for both plant species complemented the results of network robustness. These networks were obtained by first mapping transcripts from brown and green modules of *R. serpentina* and *C. roseus*, respectively, on gene-metabolite networks and further by extending these subgraphs to include first neighbors. Interestingly, the gene-metabolite network of *R. serpentina* was found to be more complex as compared to the network obtained from *C. roseus* (Figure S12). In summary, we surmise that rewiring of the interactions among candidate genes could have led to the variation in regulatory mechanisms, and thereby diversification in the late steps of TIA pathway in these closely related plants.

## Conclusions

This work comprises meta-data analysis of RNA sequencing data through identification of differentially expressed genes to compare closely related medicinal plants *R. serpentina* and *C. roseus*. The study demonstrates the utility of comparative co-expression analysis for identification of candidate genes that may be responsible for diversification of biological pathways among various species. The module preservation statistics and functional enrichment analysis revealed conservation of primary metabolite synthesis as well as that of upstream of TIA pathway. Rewiring in regulatory mechanisms of transcription factor AT2G23290 (MYB70) and two other genes (AT1G72520; LOX4, AT2G01300; unknown protein) was identified to be responsible for tissue-specific as well as species-specific synthesis of metabolites. The network robustness of *R. serpentina* network as compared to that of *C. roseus* was also complemented by complexity of its gene-metabolite network which may have evolved due to its complex metabolic mechanisms. Our study presents a strategy for identification of key genes responsible for diversification of pathways between closely related species, and thereby for revealing their evolutionary relationship.

## Author contributions

SP conceived the idea, designed and conducted the experiments, analyzed data and wrote the manuscript. GB and PA contributed to data analysis and interpretation as well as manuscript writing.

### Conflict of interest statement

The authors declare that the research was conducted in the absence of any commercial or financial relationships that could be construed as a potential conflict of interest.

## Abbreviations

DEGs: Differentially Expressed Genes
MPGR: Medicinal Plant Genomics Resource
TAIR10: The Arabidopsis Information Resource 10
PCA: Principal Component Analysis
WGCNA: Weighted Gene Co-Expression Network Analysis
GO: Gene Ontology
KEGG: Kyoto Encyclopedia of Genes and Genomes
DAVID: Database for Annotation, Visualization and Integrated Discovery
TIA: Terpenoid Indole Alkaloid
TFs: Transcription Factors.
