# Supplementary material for: Differential Network Analysis Reveals Evolutionary Complexity in Secondary Metabolism of Rauvolfia serpentina over Catharanthus roseus
Source: Front Plant Sci. 2016 Aug 18;7:1229. doi: 10.3389/fpls.2016.01229 (PMC4988974; doi:10.3389/fpls.2016.01229)
Supplement: Supplementary file 1 [file DataSheet1.PDF]

Supplementary Material

A. Supplementary Figures

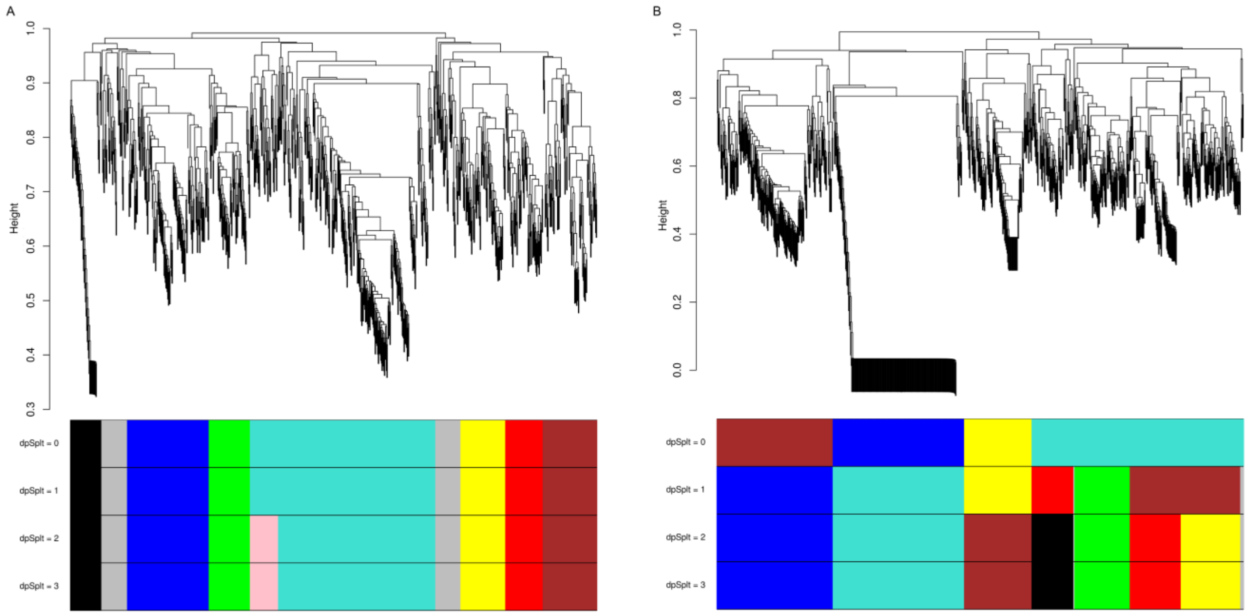

Figure S1. Gene dendrograms are constructed by average linkage hierarchical clustering for **(A)** *R. serpentina* and **(B)** *C. roseus* datasets. The color row underneath the cluster tree shows module assignment implemented by the dynamic tree cut method.

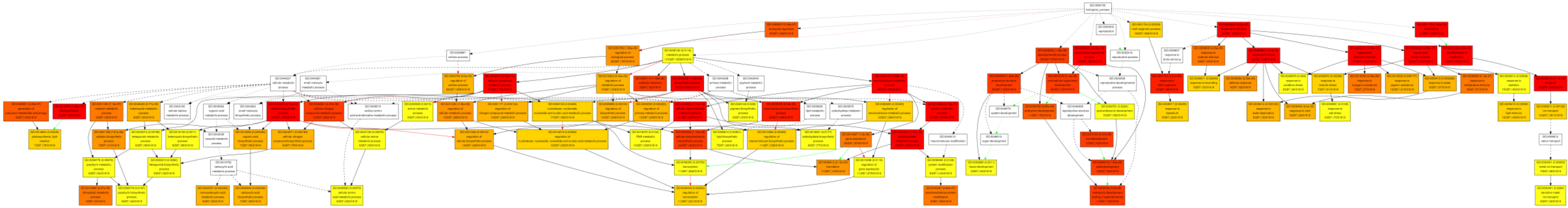

Figure S2 Hierarchical graph representing the significantly enriched GO terms for turquoise module of *R. serpentina*. These over-represented GO terms for biological process category are obtained using agriGO. Each GO term represented by box are labeled by their GO ID, term definition, and statistical information. Degree of color saturation of a box is positively correlated to the enrichment level of the term.

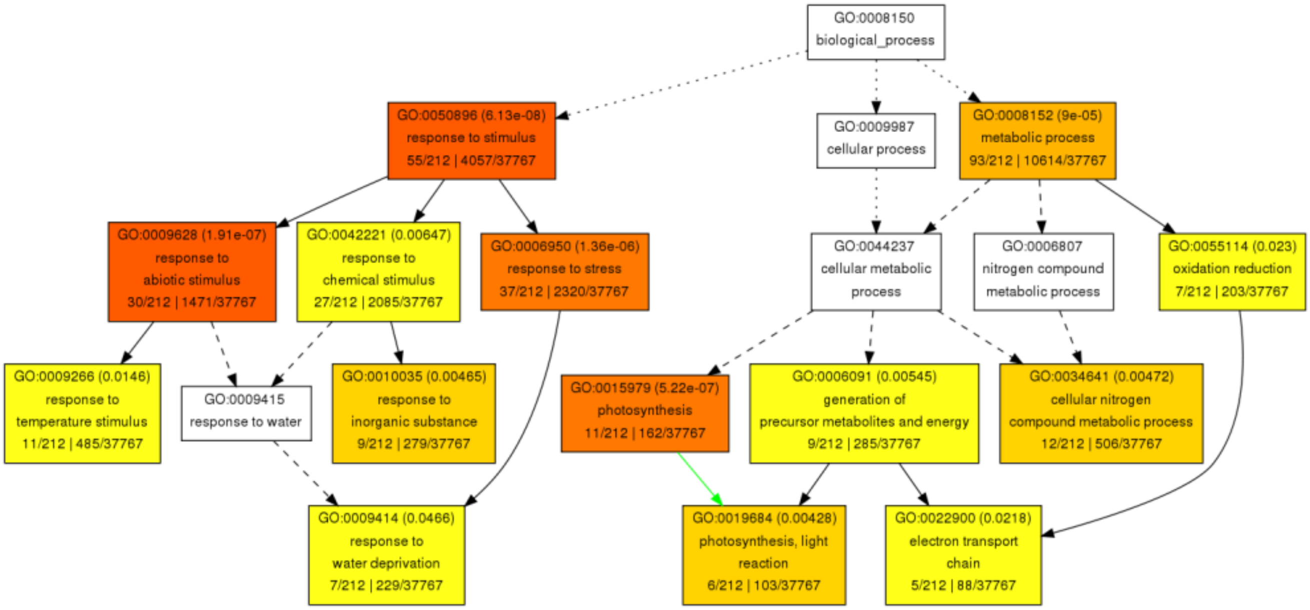

Figure S3. Hierarchical graph representing the significantly enriched GO terms for turquoise module of *C. roseus*. These over-represented GO terms for biological process category are obtained using agriGO. Each GO term represented by box are labeled by their GO ID, term definition, and statistical information. Degree of color saturation of a box is positively correlated to the enrichment level of the term.

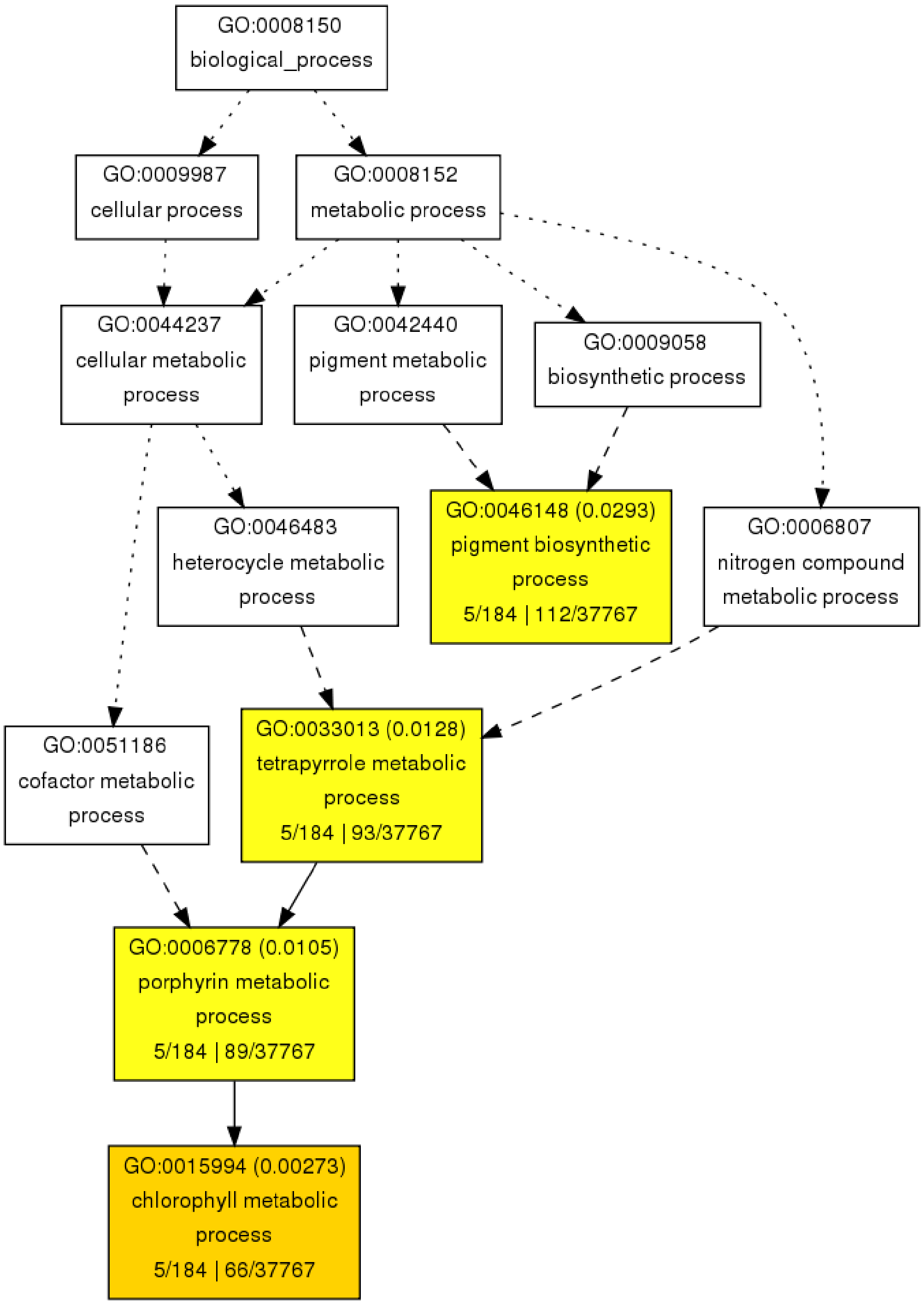

Figure S4. Hierarchical graph representing significantly enriched GO terms for blue module of *C. roseus*. These over-represented GO terms for biological process category are obtained using agriGO. Each GO term represented by box are labeled by their GO ID, term definition, and statistical information. Degree of color saturation of a box is positively correlated to the enrichment level of the term.

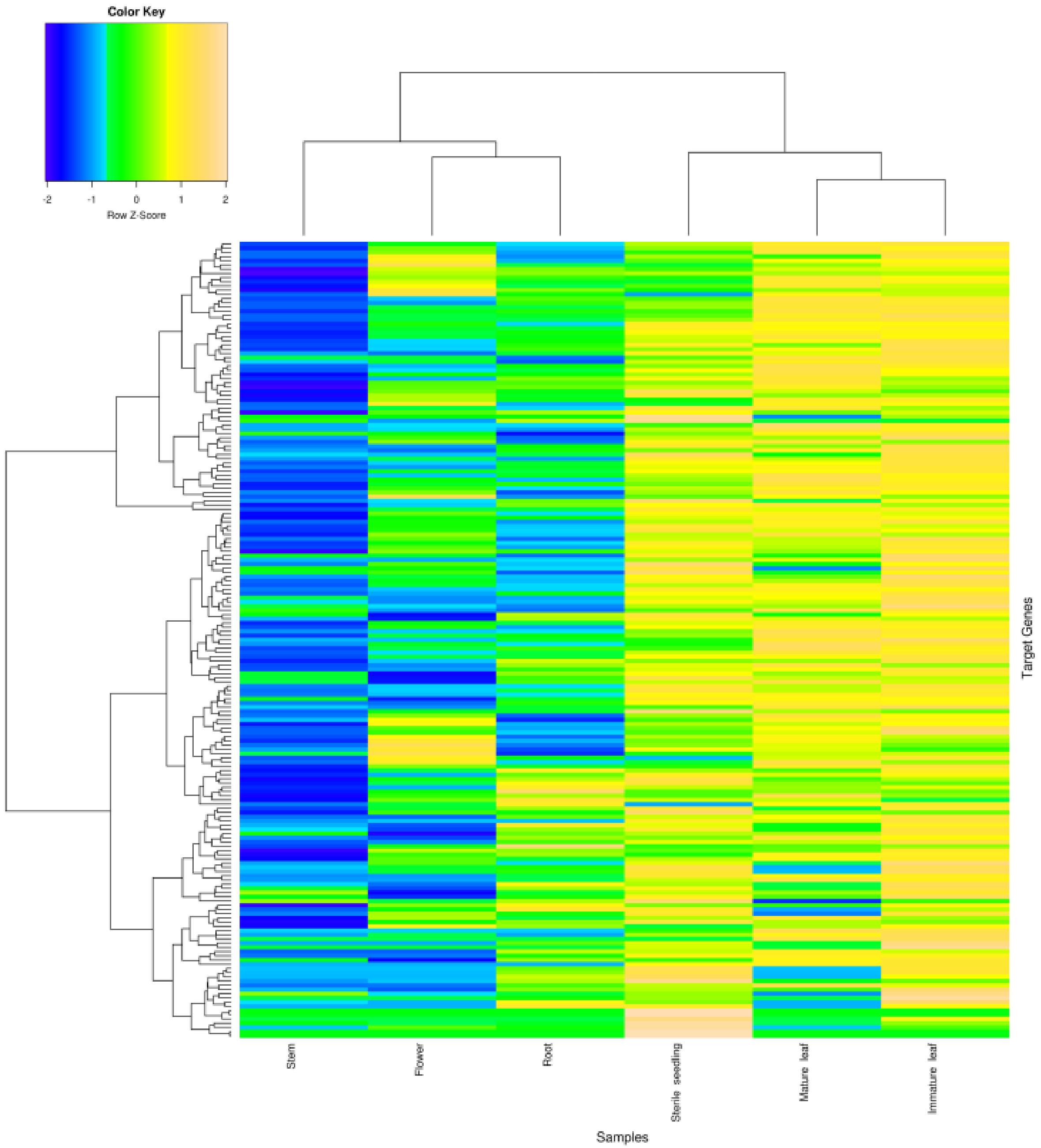

Figure S5. Heatmap of transcripts using expression data of different tissues. Heatmap is depicting tissue-specific expression of transcripts of blue module in mature and immature leaves of *C. roseus*, where average expression is calculated based on normalized transcriptomics data. The ‘gplots’ library of R package is used to plot heatmap.

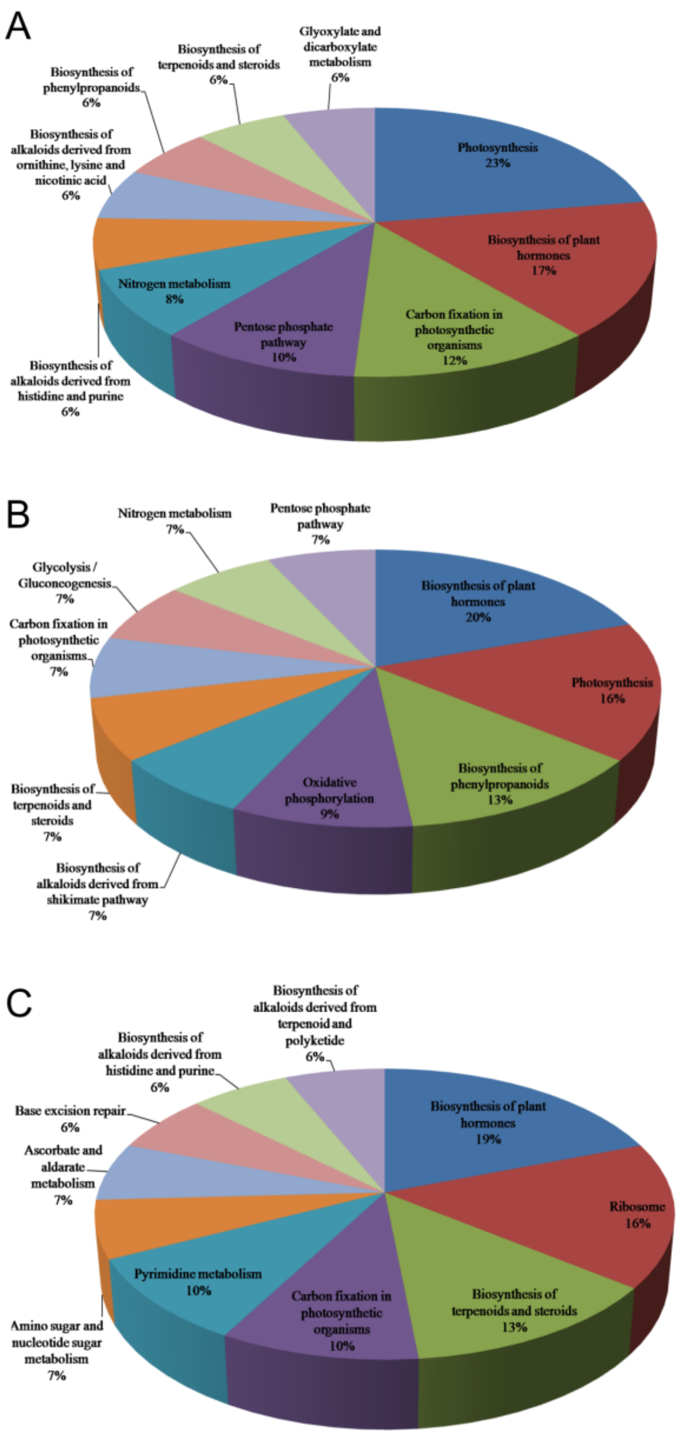

Figure S6. Pie chart representing the count of significant KEGG pathways of **(A)** turquoise module from *R. serpentina* and **(B)** turquoise as well as **(C)** blue modules from *C. roseus*, using DAVID.

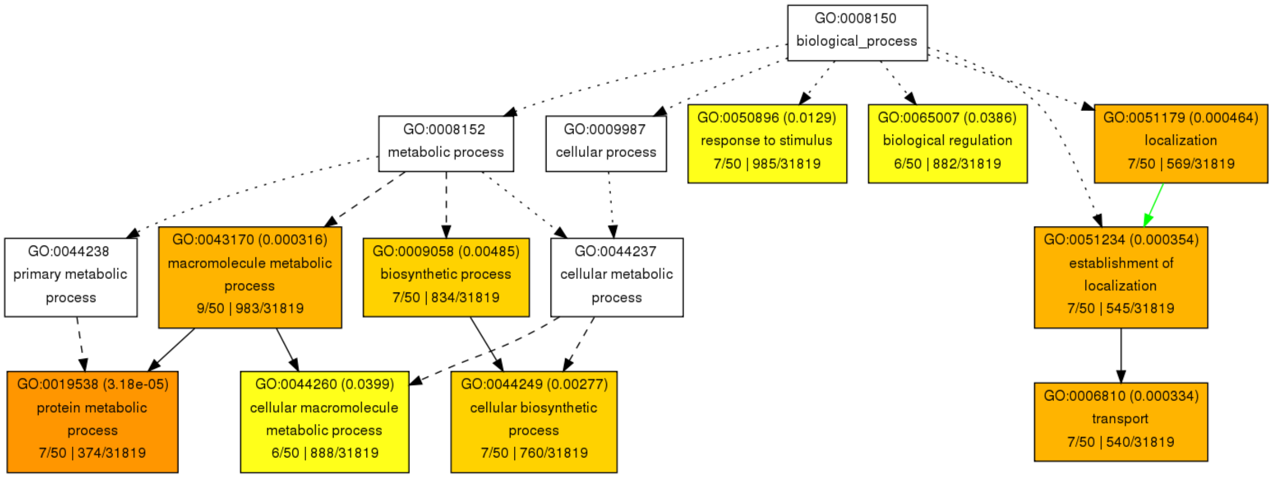

Figure S7. Hierarchical graph representing significantly enriched GO terms for black module of *R. serpentina*. These over-represented GO terms for biological process category are obtained using agriGO. Each GO term represented by box are labeled by their GO ID, term definition, and statistical information. Degree of color saturation of a box is positively correlated to the enrichment level of the term.

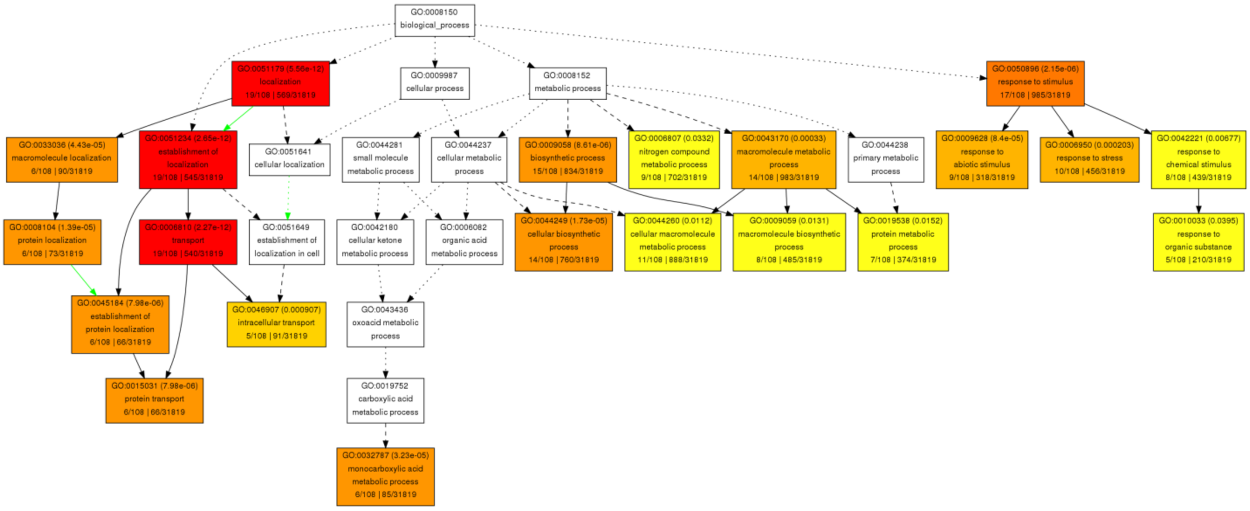

Figure S8. Hierarchical graph representing significantly enriched GO terms for brown module of *C. roseus*. These over-represented GO terms for biological process category are obtained using agriGO. Each GO term represented by box are labeled by their GO ID, term definition, and statistical information. Degree of color saturation of a box is positively correlated to the enrichment level of the term.

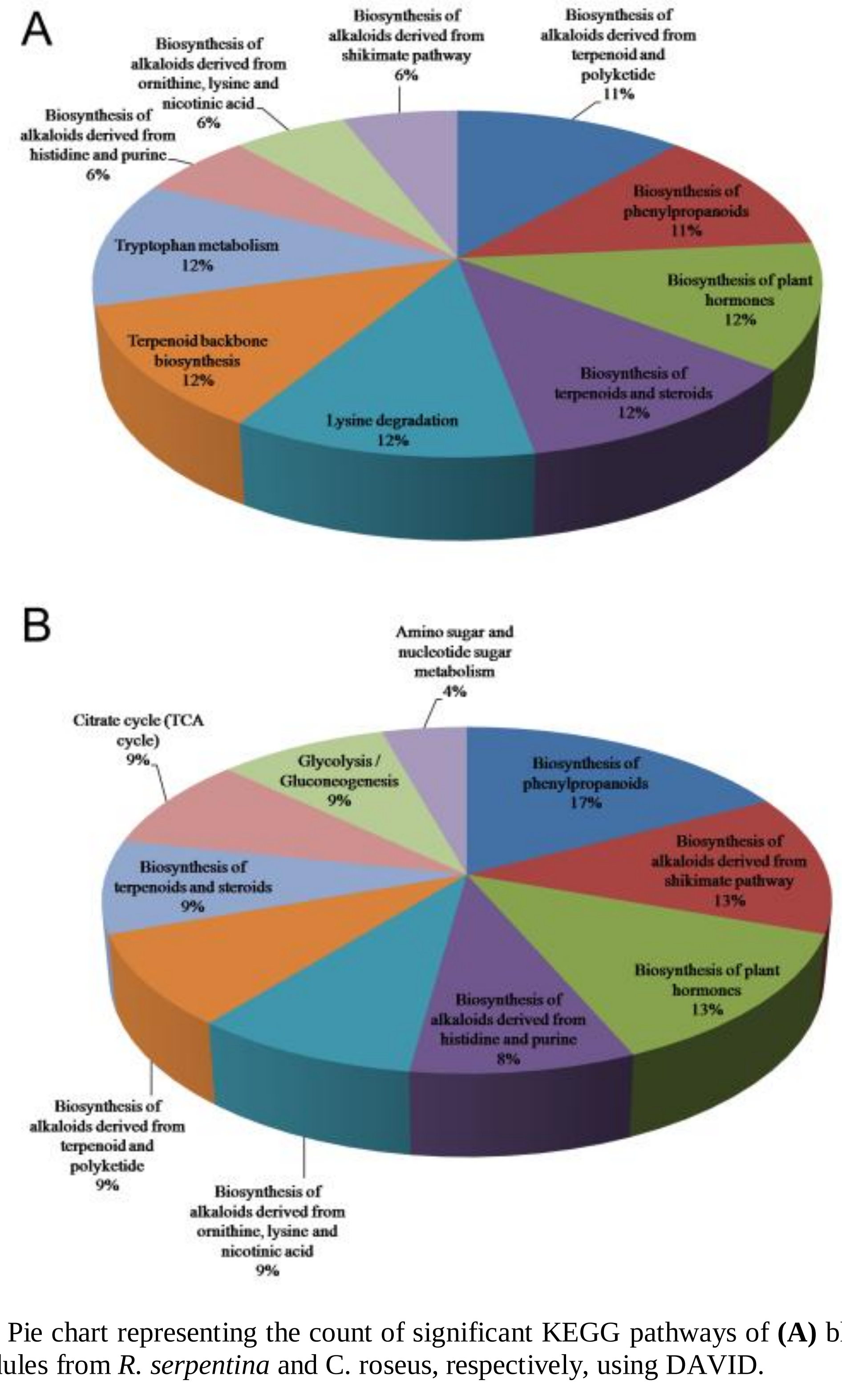

Figure S9. Pie chart representing the count of significant KEGG pathways of **(A)** black and **(B)** brown modules from *R. serpentina* and C. roseus, respectively, using DAVID.

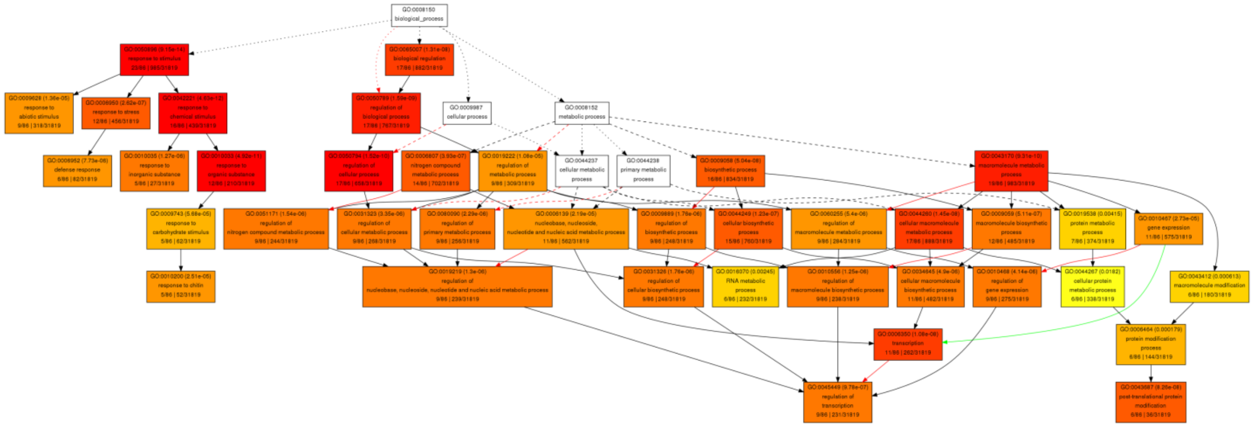

Figure S10. Hierarchical graph representing the significantly enriched GO terms for brown module of *R. serpentina*. These over-represented GO terms for biological process category are obtained using agriGO. Each GO term represented by box are labeled by their GO ID, term definition, and statistical information. Degree of color saturation of a box is positively correlated to the enrichment level of the term.

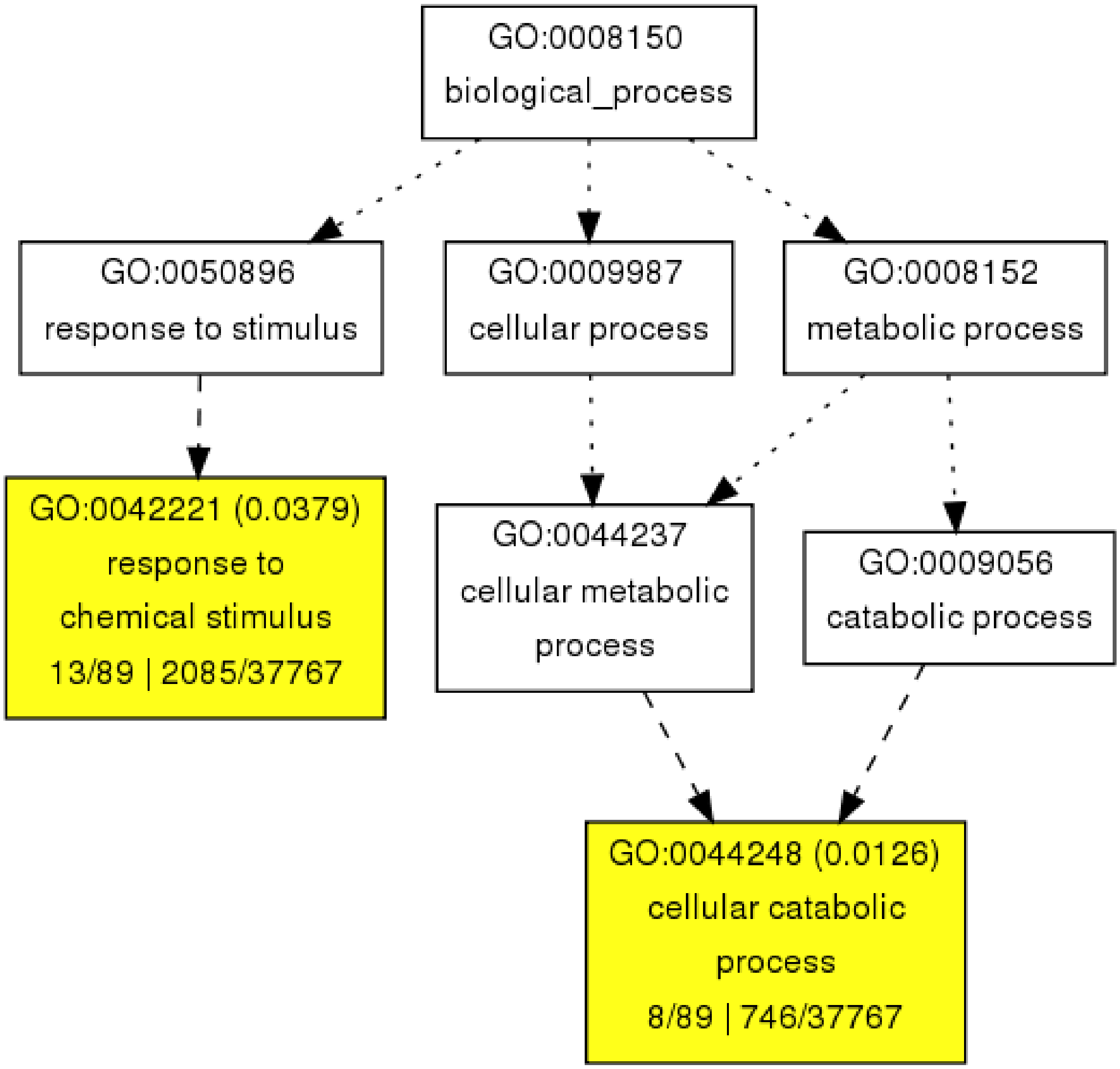

Figure S11. Hierarchical graph representing significantly enriched GO terms for green module of *C. roseus*. These over-represented GO terms for biological process category are obtained using agriGO. Each GO term represented by box are labeled by their GO ID, term definition, and statistical information. Degree of color saturation of a box is positively correlated to the enrichment level of the term.

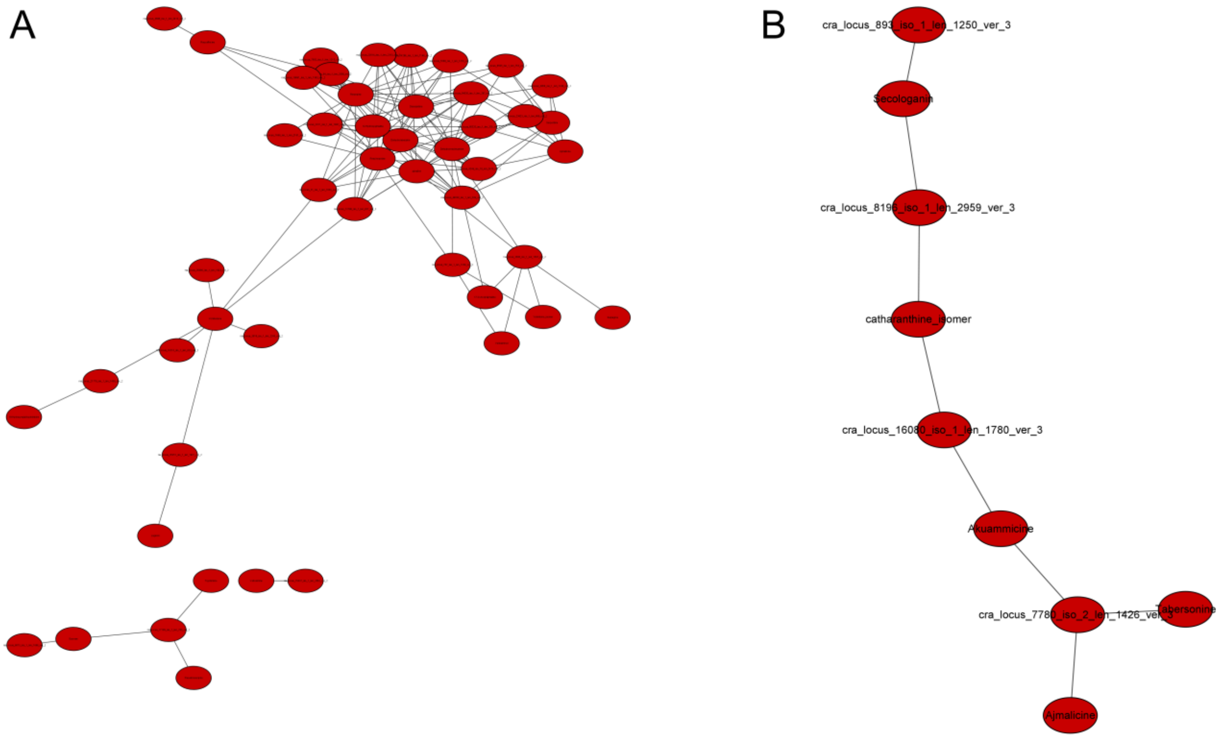

Figure S12 Gene-metabolite networks for **(A)** brown and **(B)** green modules from *R. serpentina* and *C. roseus*, respectively. Cytoscape *v* 3.1.0 program is used to generate networks.

B. Supplementary Tables

Table S1. KEGG pathway annotation of transcripts in turquoise module of *Rauvolfia serpentina* using DAVID.

S. No.	KEGG pathways	Count of Pathways
1	Photosynthesis	11
2	Biosynthesis of plant hormones	8
3	Carbon fixation in photosynthetic organisms	6
4	Pentose phosphate pathway	5
5	Nitrogen metabolism	4
6	Biosynthesis of alkaloids derived from histidine and purine	3
7	Biosynthesis of alkaloids derived from ornithine, lysine and nicotinic acid	3
8	Biosynthesis of phenylpropanoids	3
9	Biosynthesis of terpenoids and steroids	3
10	Glyoxylate and dicarboxylate metabolism	3
11	Oxidative phosphorylation	3
12	Porphyrin and chlorophyll metabolism	3
13	Pyrimidine metabolism	3
14	Ribosome	3
15	Alanine, aspartate and glutamate metabolism	2
16	alpha-Linolenic acid metabolism	2
17	Arachidonic acid metabolism	2
18	Biosynthesis of alkaloids derived from shikimate pathway	2
19	Fructose and mannose metabolism	2
20	Glutathione metabolism	2
21	Pentose and glucuronate interconversions	2
22	Purine metabolism	2
23	Aminoacyl-tRNA biosynthesis	1
24	Amino sugar and nucleotide sugar metabolism	1
25	Arginine and proline metabolism	1
26	Ascorbate and aldarate metabolism	1
27	Base excision repair	1
28	Biosynthesis of alkaloids derived from terpenoid and polyketide	1
29	Biosynthesis of unsaturated fatty acids	1
30	Carotenoid biosynthesis	1
31	Citrate cycle (TCA cycle)	1
32	Cyanoamino acid metabolism	1
33	Cysteine and methionine metabolism	1
34	DNA replication	1
35	Folate biosynthesis	1
36	Glycine, serine and threonine metabolism	1
37	Glycolysis / Gluconeogenesis	1

38	Homologous recombination	1
39	Limonene and pinene degradation	1
40	Methane metabolism	1
41	Mismatch repair	1
42	Nucleotide excision repair	1
43	Proteasome	1
44	Pyruvate metabolism	1
45	RNA degradation	1
46	Selenoamino acid metabolism	1
47	Starch and sucrose metabolism	1
48	Stilbenoid, diarylheptanoid and gingerol biosynthesis	1
49	Sulfur metabolism	1
50	Tropane, piperidine and pyridine alkaloid biosynthesis	1
51	Ubiquitin mediated proteolysis	1
52	Valine, leucine and isoleucine degradation	1
53	Vitamin B6 metabolism	1

Table S2. KEGG pathway annotation of transcripts in turquoise module of *Catharanthus roseus* using DAVID.

S. No.	KEGG pathways	Count of Pathways
1	Biosynthesis of plant hormones	11
2	Photosynthesis	9
3	Biosynthesis of phenylpropanoids	7
4	Oxidative phosphorylation	5
5	Biosynthesis of alkaloids derived from shikimate pathway	4
6	Biosynthesis of terpenoids and steroids	4
7	Carbon fixation in photosynthetic organisms	4
8	Glycolysis / Gluconeogenesis	4
9	Nitrogen metabolism	4
10	Pentose phosphate pathway	4
11	Ribosome	4
12	alpha-Linolenic acid metabolism	3
13	Biosynthesis of alkaloids derived from histidine and purine	3
14	Biosynthesis of alkaloids derived from terpenoid and polyketide	3
15	Valine, leucine and isoleucine degradation	3
16	Alanine, aspartate and glutamate metabolism	2
17	Arachidonic acid metabolism	2
18	Biosynthesis of alkaloids derived from ornithine, lysine and nicotinic acid	2
19	Fatty acid metabolism	2
20	Fructose and mannose metabolism	2
21	Glutathione metabolism	2
22	Glycine, serine and threonine metabolism	2
23	Phenylalanine, tyrosine and tryptophan biosynthesis	2
24	Purine metabolism	2
25	Spliceosome	2
26	Terpenoid backbone biosynthesis	2
27	Tryptophan metabolism	2
28	Aminoacyl-tRNA biosynthesis	1
29	Amino sugar and nucleotide sugar metabolism	1
30	Arginine and proline metabolism	1
31	Ascorbate and aldarate metabolism	1
32	Biosynthesis of unsaturated fatty acids	1
33	Butanoate metabolism	1
34	Carotenoid biosynthesis	1
35	Cyanoamino acid metabolism	1
36	Cysteine and methionine metabolism	1
37	Endocytosis	1
38	Flavonoid biosynthesis	1
39	Galactose metabolism	1

40	Glyoxylate and dicarboxylate metabolism	1
41	Inositol phosphate metabolism	1
42	Limonene and pinene degradation	1
43	Lysine degradation	1
44	Methane metabolism	1
45	N-Glycan biosynthesis	1
46	One carbon pool by folate	1
47	Phenylpropanoid biosynthesis	1
48	Porphyrin and chlorophyll metabolism	1
49	Propanoate metabolism	1
50	Proteasome	1
51	Pyrimidine metabolism	1
52	Pyruvate metabolism	1
53	Selenoamino acid metabolism	1
54	Starch and sucrose metabolism	1
55	Steroid biosynthesis	1
56	Stilbenoid, diarylheptanoid and gingerol biosynthesis	1
57	Synthesis and degradation of ketone bodies	1
58	Valine, leucine and isoleucine biosynthesis	1

Table S3. KEGG pathway annotation of transcripts in blue module of *Catharanthus roseus* using DAVID.

S. No.	KEGG pathways	Count of Pathways
1	Biosynthesis of plant hormones	6
2	Ribosome	5
3	Biosynthesis of terpenoids and steroids	4
4	Carbon fixation in photosynthetic organisms	3
5	Pyrimidine metabolism	3
6	Amino sugar and nucleotide sugar metabolism	2
7	Ascorbate and aldarate metabolism	2
8	Base excision repair	2
9	Biosynthesis of alkaloids derived from histidine and purine	2
10	Biosynthesis of alkaloids derived from terpenoid and polyketide	2
11	DNA replication	2
12	Nucleotide excision repair	2
13	Pentose phosphate pathway	2
14	Photosynthesis	2
15	Porphyrin and chlorophyll metabolism	2
16	Purine metabolism	2
17	Pyruvate metabolism	2
18	Starch and sucrose metabolism	2
19	alpha-Linolenic acid metabolism	1
20	Biosynthesis of alkaloids derived from ornithine, lysine and nicotinic acid	1
21	Biosynthesis of alkaloids derived from shikimate pathway	1
22	Biosynthesis of phenylpropanoids	1
23	Biosynthesis of unsaturated fatty acids	1
24	Carotenoid biosynthesis	1
25	Citrate cycle (TCA cycle)	1
26	Cyanoamino acid metabolism	1
27	Cysteine and methionine metabolism	1
28	Fatty acid biosynthesis	1
29	Folate biosynthesis	1
30	Fructose and mannose metabolism	1
31	Galactose metabolism	1
32	Glyoxylate and dicarboxylate metabolism	1
33	Mismatch repair	1
34	Nitrogen metabolism	1
35	Pentose and glucuronate interconversions	1
36	Propanoate metabolism	1
37	RNA degradation	1
38	Selenoamino acid metabolism	1
39	Sulfur metabolism	1

40	Terpenoid backbone biosynthesis	1
41	Ubiquitin mediated proteolysis	1
42	Vitamin B6 metabolism	1

Table S4. KEGG pathway annotation of transcripts in black module of *Rauvolfia serpentina* using DAVID.

S. No.	KEGG pathways	Count of Pathways
1	Biosynthesis of alkaloids derived from terpenoid and polyketide	2
2	Biosynthesis of phenylpropanoids	2
3	Biosynthesis of plant hormones	2
4	Biosynthesis of terpenoids and steroids	2
5	Lysine degradation	2
6	Terpenoid backbone biosynthesis	2
7	Tryptophan metabolism	2
8	Biosynthesis of alkaloids derived from histidine and purine	1
9	Biosynthesis of alkaloids derived from ornithine, lysine and nicotinic acid	1
10	Biosynthesis of alkaloids derived from shikimate pathway	1
11	Biosynthesis of unsaturated fatty acids	1
12	Butanoate metabolism	1
13	Citrate cycle (TCA cycle)	1
14	Fatty acid metabolism	1
15	Flavone and flavonol biosynthesis	1
16	Galactose metabolism	1
17	Oxidative phosphorylation	1
18	Phenylpropanoid biosynthesis	1
19	Propanoate metabolism	1
20	Proteasome	1
21	Pyruvate metabolism	1
22	Starch and sucrose metabolism	1
23	Synthesis and degradation of ketone bodies	1
24	Valine, leucine and isoleucine degradation	1

Table S5. KEGG pathway annotation of transcripts in brown module of *Catharanthus roseus* using DAVID.

S. No.	KEGG pathways	Count of Pathways
1	Biosynthesis of phenylpropanoids	4
2	Biosynthesis of alkaloids derived from shikimate pathway	3
3	Biosynthesis of plant hormones	3
4	Biosynthesis of alkaloids derived from histidine and purine	2
5	Biosynthesis of alkaloids derived from ornithine, lysine and nicotinic acid	2
6	Biosynthesis of alkaloids derived from terpenoid and polyketide	2
7	Biosynthesis of terpenoids and steroids	2
8	Citrate cycle (TCA cycle)	2
9	Glycolysis / Gluconeogenesis	2
10	Amino sugar and nucleotide sugar metabolism	1
11	Ascorbate and aldarate metabolism	1
12	Basal transcription factors	1
13	Carbon fixation in photosynthetic organisms	1
14	Diterpenoid biosynthesis	1
15	Fatty acid biosynthesis	1
16	Flavone and flavonol biosynthesis	1
17	Fructose and mannose metabolism	1
18	Galactose metabolism	1
19	Glyoxylate and dicarboxylate metabolism	1
20	Inositol phosphate metabolism	1
21	Phenylalanine, tyrosine and tryptophan biosynthesis	1
22	Phenylpropanoid biosynthesis	1
23	Porphyrin and chlorophyll metabolism	1
24	Pyruvate metabolism	1
25	Starch and sucrose metabolism	1

Table S6. KEGG pathway annotation of transcripts in brown module of *Rauvolfia serpentina* using DAVID.

S. No.	KEGG pathways	Count of Pathways
1	Biosynthesis of plant hormones	2
2	alpha-Linolenic acid metabolism	1
3	Biosynthesis of alkaloids derived from terpenoid and polyketide	1
4	Biosynthesis of terpenoids and steroids	1
5	Nitrogen metabolism	1
6	Phosphatidylinositol signaling system	1
7	Spliceosome	1
8	Terpenoid backbone biosynthesis	1

Table S7. KEGG pathway annotation of transcripts in green module of *Catharanthus roseus* using DAVID.

S. No.	KEGG pathways	Count of Pathways
1	Biosynthesis of plant hormones	4
2	Biosynthesis of alkaloids derived from ornithine, lysine and nicotinic acid	3
3	Biosynthesis of alkaloids derived from shikimate pathway	3
4	Biosynthesis of phenylpropanoids	3
5	Oxidative phosphorylation	2
6	alpha-Linolenic acid metabolism	1
7	Arginine and proline metabolism	1
8	Biosynthesis of alkaloids derived from histidine and purine	1
9	Biosynthesis of alkaloids derived from terpenoid and polyketide	1
10	Biosynthesis of terpenoids and steroids	1
11	Citrate cycle (TCA cycle)	1
12	Homologous recombination	1
13	Lysine degradation	1
14	Nitrogen metabolism	1
15	Pentose and glucuronate interconversions	1
16	Pentose phosphate pathway	1
17	Phenylalanine metabolism	1
18	Phenylpropanoid biosynthesis	1
19	Phosphatidylinositol signaling system	1
20	Proteasome	1
21	Regulation of autophagy	1
22	Tropane, piperidine and pyridine alkaloid biosynthesis	1
23	Tryptophan metabolism	1
